# A flexible exponential type family for modeling non-monotonic hazard rates with application to mortality analysis: A COVID-19 case study

**DOI:** 10.1371/journal.pone.0331050

**Published:** 2026-08-06

**Authors:** Abdulrahman Obaid Alshammari, Hadi Obaid Alshammari, Hameed Ali, Bilal Himmat

**Affiliations:** 1 Department of Mathematics, College of Science, Jouf University, Sakaka, Saudi Arabia; 2 Higher Education Archives and Libraries Department of Khyber Pakhtunkhwa, Pakistan; 3 Department of Computer Science, Faculty of Computer Science, Kabul University (KU), Kabul, Afghanistan; Universita degli Studi di Milano, ITALY

## Abstract

This paper aiming at the formulation of a parsimonious two-parameter family of lifetime distributions obtained by a simple but flexible exponential-type transformation of an arbitrary baseline CDF (the Flexible Exponential-Type Family, FETF) and study in detail its Exponential-Type Weibull (ETW) special case. The ETW retains analytical tractability while permitting bathtub-shaped and unimodal hazard rate behavior frequently observed in epidemiology and reliability. To theoretically validate the model, we derive key distributional properties, including the probability density function, survival function, and hazard rate. We present maximum-likelihood estimation (MLE): score equations, observed Fisher information, and asymptotic confidence intervals. We address requirements that guarantee negative definiteness and provide explicit formulas for the Hessian (observed information). Monte Carlo simulations evaluating bias, mean squared error, and model selection frequency as well as application to COVID-19 data (n = 106) from Mexican public records are used to empirically validate the performance of the proposed ETW distribution. The ETW consistently performs better than well-known competing models, such as the Weibull and other state-of-the-art distributions, across information requirements and goodness-of-fit metrics. For simulating non-monotonic hazard issues in epidemiology and reliability investigations, the ETW distribution provides a flexible and computationally easy tool.

## 1. Introduction

**Fig 1:**
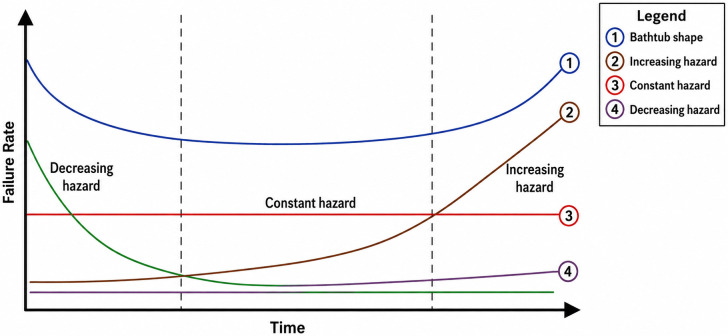
Graphical illustration of representative hazard rate patterns, including a bathtub-shaped hazard, monotonically increasing hazard, monotonically decreasing hazard, and a constant hazard function.

The COVID-19 pandemic, which was brought on by the new SARS-CoV-2 virus, has put tremendous strain on healthcare systems and brought attention to the need for precise statistical modeling in order to comprehend and forecast mortality trends. [1]. Empirical hazard trajectories frequently display complex, non-monotonic behavior that cannot be handled by traditional parametric families, such as exponential, Weibull, and log-normal, which offer straightforward, understandable expressions for the hazard. Non-monotonic risks can occur in a variety of situations, such as short-term peaks linked to acute exposure and longer-term drops as vulnerable populations are reduced, bathtub-shaped patterns in reliability contexts, or initial, brief rises in risk followed by declines [2]. In order to accurately describe the underlying process and to make trustworthy predictions and policy inferences, it is crucial to capture these intricate danger structures. Although there are adaptable semi- and nonparametric tools, they could compromise interpretability, closed-form expressions, or simple likelihood-based inferential procedures [3,4]. On the other hand, parametric expansions that maintain tractability while significantly expanding the range of acceptable hazard shapes might be very appealing; they allow likelihood-based estimate, maintain succinct summaries, and easily incorporate into regression or hierarchical models. Because the exponential distribution assumes a constant hazard rate, it is not as useful in real-world situations where risk varies over time [5]. As seen in Fig 1, the Weibull distribution expands on the exponential model by allowing monotone hazard trajectories that are either increasing or decreasing. However, it is intrinsically unsuitable to depict non-monotonic hazard profiles, such as bathtub-shaped or unimodal dangers, which frequently occur in epidemics and other complex systems.

The literature offers a wide range of generalizations and novel distributional families to get around these restrictions. Many changes and generalized families have been suggested to improve the flexibility of the Weibull model. Early approaches include modified-Weibull [6] and the Beta-Transmuted Weibull construction [7], which introduce additional shape parameters to broaden the class of attainable hazard forms. Subsequent research has yielded increasingly comprehensive distributional frameworks, including the T–X family [8], the modified Weibull family [9], the exponential class [10], and Alpha-Power transformations, all of which have laid a systematic methodological basis for enhancing distributional flexibility. Although highly parameterized distributions, such as the five-parameter Exponentiated Weibull distribution [11] and the Exponentiatial-type Logistic Power Generalized Weibull distribution [1], provide considerable shape adaptability, they do so at the expense of increased computational complexity and a heightened susceptibility to overfitting.

In response, several parsimonious yet flexible distribution families such as Kumaraswamy family [12], the odd N–H family [13], M-family variants [14], the transmuted Fréchet family [15], the exponentiated Alpha-Power family [16], the Cauchy Power Weibull and Perks families [17], and modified Weibull-type distribution [18] have been introduced to balance interpretability with modeling versatility.

Motivated by the limitations of existing models in capturing complex, non-monotonic hazard structures observed in COVID-19 mortality data, we develop a new Flexible Exponential-Type Family (FETF) of distributions. As a special case, the ETW distribution constitutes a two-parameter parsimonious distribution capable of accommodating both bathtub-shaped and unimodal hazard functions, while maintaining analytical tractability and interpretability. As a result, the ETW provides a useful compromise between adequate shape flexibility and structural simplicity for accurate lifespan modeling. The Modified Alpha-Power Weibull [17,19–21], the Type-I Cosine Exponentiated Weibull [22], and other recent distributional contributions [23–28] are recent related modeling inventions that show comparable goals and performance.

By applying a change to the cumulative hazard structure of a baseline parametric model, we present a unique exponential-type family of distributions in this study. The architecture is purposefully frugal, retaining many analytical advantages of the original baseline distribution while adding shape flexibility through a few extra parameters. We define and thoroughly examine the Exponential-Type Weibull (ETW) distribution as a specific and instructive particular instance. Depending on the parameter setup, the ETW accepts non-monotonic hazard functions, such as unimodal and bathtub forms, while maintaining the Weibull’s well-known scale-shape parameterization. To understand how the additional parameters govern hazard curvature and tail behavior, we calculate the density, survivor, and hazard functions, get expressions for moments when available, and analyze the limiting behavior.

The remaining paper’s structure falls under the following categories:

The FETF family is formalized in Section 2. Section 3 derives the ETW distribution. Section 4 establishes its mathematical properties. Section 5 details MLE theory. Section 6 computes Shannon entropy. Section 7 present empirical and simulation results. Section 8 concludes.

## 2. FETF of distributions

Let *X* denote a continuous random variable. The cumulative distribution function (CDF) for this newly defined FETF family is expressed as:


$$\begin{array}{c} F_{FETF}(x)=\left\{ \begin{array}{c} exp\left(\Phi \left(\text{1}-\frac{\text{1}}{F(x)}\right)\right);if\Phi >\text{1}\\ exp\left(\text{1}-\frac{\text{1}}{F(x)}\right)\;;if\Phi =\text{1} \end{array}\right. \end{array}$$ (1)

Here, the parameter Φ governs scale, while F(x) denotes the baseline cumulative distribution function.

### 2.1. Formulation of the ETW distribution

We now introduce a key submodel of the FETF framework. By appropriately modifying the cumulative distribution function of the classical Weibull distribution, we derive a new cumulative distribution function that defines the Exponential-Type Weibull (ETW) distribution, as expressed in Equation (3). The Weibull distribution CDF is formulated as follows.


$$\begin{array}{c} F_{w}(x)=\text{1}-exp\left(-\Phi x^{\tau }\right),x>\text{0} \end{array}$$ (2)

Where, Φ and τ represent the scale and shape parameter, respectively.


$$\begin{array}{c} F_{ETW}(x)=exp\left(\Phi \left(\text{1}-\frac{\text{1}}{\text{1}-e^{-\Phi x^{\tau }}}\right)\right);x>\text{0}\&\Phi ,\tau >\text{0} \end{array}$$ (3)

The probability density function (PDF) associated with Equation (3) can be displayed as follows:


$$\begin{array}{c} f_{ETW}(x)=\frac{\Phi^{\text{2}}\tau x^{\tau -\text{1}}exp\left(\Phi x^{\tau }-\frac{\Phi }{e^{\Phi x^{\tau }}-\text{1}}\right)}{(exp\left(\Phi x^{\tau }\right)-\text{1}{)}^{\text{2}}};x>\text{0}\&\Phi ,\tau >\text{0} \end{array}$$ (4)

The ETW is a two-parameter distribution where both Φ and τ are shape parameters. The parameter τ controls the basic shape akin to the Weibull distribution, while Φ, the generator parameter, provides the additional flexibility needed to model non-monotonic hazards.

**Fig 2:**
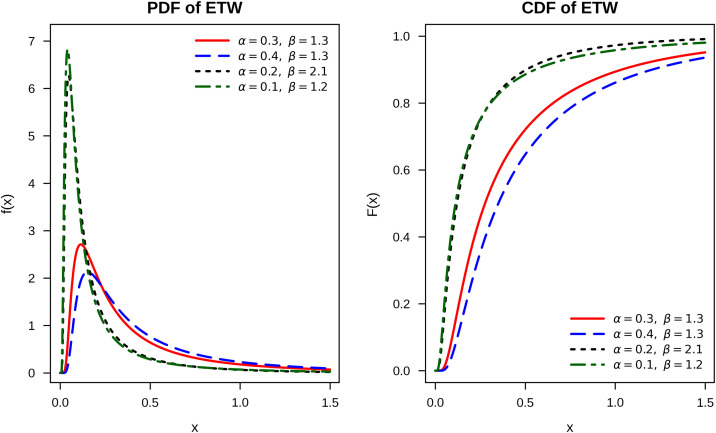
Probability density and cumulative distribution functions of the ETW distribution for selected parameter values.

The pictorial representation of the ETW PDF and the CDF for various parameter values is shown in Fig 2. The Flexible Exponential-Type Family (FETF) is proposed to generate new distributions with enhanced flexibility in their hazard rate functions. The transformation $\text{1}-\frac{\text{1}}{F(x)}$ within the exponential function is designed to effectively introduce non-monotonicity into the hazard function of the baseline distribution. This specific form was chosen empirically and through comparative analysis, as it consistently produces a rich variety of hazard shapes, including the unimodal and bathtub shapes that are the focus of this paper, while maintaining a degree of mathematical tractability for deriving statistical properties.

## 3. Statistical properties

This section examines the principal statistical properties of the ETW distribution. For important features, such as the survival and hazard functions, moments, quantile function, mean residual life, order statistics, and Shannon entropy, we construct analytical formulas (or tractable series representations when needed). These results establish the theoretical foundation required for inference and empirical application of the model.

### 3.1. The survival and hazard rate function

Let X be a random variable follows ETW(Φ,τ), then the survival function of the proposed distribution is defined as R(x)=1−F(x), substituting into Equation (3), we have


$$\begin{array}{c} R(x)=\text{1}-\text{exp}\left(\Phi \left[\text{1}-\frac{\text{1}}{\text{1}-exp\left(-\Phi x^{\tau }\right)}\right]\right), \end{array}$$

Or


$$\begin{array}{c} R(x)=\text{1}-\text{exp}\left(\frac{\Phi }{{\text{exp}(}^{\Phi x^{\tau }}-\text{1}}\right). \end{array}$$ (5)

Equation (5) is the survival function of the ETW, and the hazard function can be defined as


$$\begin{array}{c} H(x)=\frac{f(x)}{R(x)}. \end{array}$$

Substituting into Equation (4) and (5) in Equation (6), we have


$$\begin{array}{c} H(x)=\frac{\Phi^{\text{2}}\tau x^{\tau -\text{1}}\text{exp}\left(\Phi x^{\tau }-\frac{\Phi }{\text{exp}\left(\Phi x^{\tau }\right)-\text{1}}\right)}{(\text{1}-\text{exp}\left(-\frac{\Phi }{\text{exp}\left(\Phi x^{\tau }\right)-\text{1}}\right))(\text{exp}\left(\Phi x^{\tau }\right)-\text{1}{)}^{\text{2}}}. \end{array}$$ (6)

After algebraic simplification, the hazard function can be expressed in a closed-form representation.


$$\begin{array}{c} H(x)=\frac{\Phi^{\text{2}}\tau x^{\tau -\text{1}}\text{exp}\left(\Phi x^{\tau }\right)}{(\text{exp}\left(\frac{\Phi }{\text{exp}\left(\Phi x^{\tau }\right)-\text{1}}\right)-\text{1})(\text{exp}\left(\Phi x^{\tau }\right)-\text{1}{)}^{\text{2}}}. \end{array}$$ (7)

**Fig 3:**
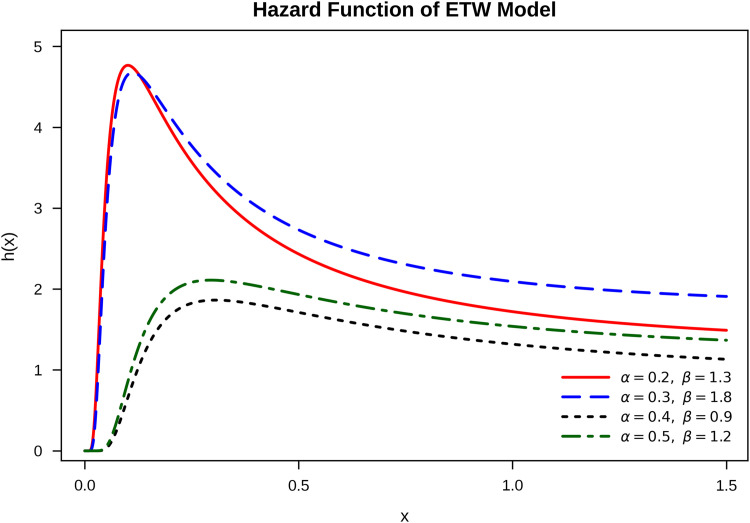
Hazard Function of ETW with examples of bathtub (Φ = 0.5, τ = 0.8) and unimodal (Φ = 2.5, τ = 2.5) shapes.

Fig 3 shows how the hazard rate function varies for various parameter values.

### 3.2. Mean residual life (MRL) function

The MRL of the ETW distribution with parameter Φ, τ is defined as


$$\begin{array}{c} M_{R}(t)=\frac{\int_{t}^{\infty }xf(x)dx}{R(x)}-t. \end{array}$$ (8)

Where f(x) and R(x) are the PDF and survival function given in Equations (4) and (5), respectively. Substituting in (8), we have


$$\begin{array}{c} M_{R}(t)=\frac{\int_{t}^{\infty }x\frac{\Phi^{\text{2}}\tau x^{\tau -\text{1}}\text{exp}\left(\Phi x^{\tau }-\frac{\Phi }{\text{exp}\left(\Phi x^{\tau }\right)-\text{1}}\right)}{{\left(\text{exp}\left(\Phi x^{\tau }\right)-\text{1}\right)}^{\text{2}}}dx}{\text{1}-\text{exp}\left(\frac{\Phi }{\text{exp}\left(\Phi x^{\tau }\right)-\text{1}}\right)}-t, \end{array}$$ (9)

First, we solve the numerator of Equation (9),


$$\begin{array}{c} Numerator=\int_{t}^{\infty }x\frac{\Phi^{\text{2}}\tau x^{\tau -\text{1}}\text{exp}\left(\Phi x^{\tau }-\frac{\Phi }{\text{exp}\left(\Phi x^{\tau }\right)-\text{1}}\right)}{{\left(\text{exp}\left(\Phi x^{\tau }\right)-\text{1}\right)}^{\text{2}}}dx, \end{array}$$ (10)

Let $z=\frac{\Phi }{\text{exp}\left(\Phi x^{\tau }\right)-\text{1}}$, $dz=-\frac{\Phi^{\text{2}}\tau x^{\tau -\text{1}}\text{exp}\left(\Phi x^{\tau }\right)}{(\text{exp}\left(\Phi x^{\tau }\right)-\text{1}{)}^{\text{2}}}dx$ and $x={\left(\frac{log(\Phi /\text{2}+\text{1})}{\Phi }\right)}^{\frac{\text{1}}{\tau }}$

Putting these three expressions in (10), we obtain:


$$\begin{array}{c} {\text{Numerator }=\left(\frac{\text{1}}{\Phi }\right)}^{\frac{\text{1}}{\tau }}\int_{\text{0}}^{\frac{\Phi }{\text{exp}\left(\Phi t^{\tau }\right)-\text{1}}}{\left(log\left(\frac{\Phi }{z}+\text{1}\right)\right)}^{\frac{\text{1}}{\tau }}\text{exp}(-z)dz, \end{array}$$ (11)

Where, ${\left(log\left(\frac{\Phi }{z}+\text{1}\right)\right)}^{\frac{\text{1}}{\tau }}={\left(-\sum_{k=\text{1}}^{\infty }\frac{(-\text{1}{)}^{k}(\Phi /z{)}^{k}}{k}\right)}^{\frac{\text{1}}{\tau }}$ for $\left|\frac{\Phi }{z}\right|<\text{1}$ putting in Equation (11), we obtain:


$$\begin{array}{c} Numerator={\left(\frac{\text{1}}{\Phi }\right)}^{\frac{\text{1}}{\tau }}{\left(-\sum_{k=\text{1}}^{\infty }\frac{(-\text{1}{)}^{k}}{k}\right)}^{\frac{\text{1}}{\tau }}\Phi^{\frac{k}{\tau }}\Gamma \left((-k-\tau \;)/\;\tau )\;,Z)\;\right), \end{array}$$ (12)

It was assumed that τ(k−2τ)≠0,τ(k−τ)≠0.

After simplification and substituting Equation (12) in (9), we obtained the MRL in the form given by:


$$\begin{array}{c} M_{R}(t)=\frac{{\left(\frac{\text{1}}{\Phi }\right)}^{\frac{\text{1}}{\tau }}{\left(-\sum_{k=\text{1}}^{\infty }\frac{(-\text{1}{)}^{k}}{k}\right)}^{\frac{\text{1}}{\tau }}\Phi^{\frac{k}{\tau }}\Gamma \left((-k-\tau \;)/\;\tau )\;,Z)\;\right)}{\text{1}-t-\text{exp}(z)}. \end{array}$$

### 3.3. The r^th^ moments

Consider a random variable X following the ETW, and then the rth moments about the origin are defined as follows:


$$\begin{array}{c} \mu_{r}=\int_{\text{0}}^{\infty }x^{r}f(x)dx, \end{array}$$

Substituting into Equation (4), we obtain:


$$\begin{array}{c} \mu_{r}=\int_{\text{0}}^{\infty }x^{r}\frac{\Phi^{\text{2}}\tau x^{\tau -\text{1}}\text{exp}\left(\Phi x^{\tau }-\frac{\Phi }{\text{exp}\left(\Phi x^{\tau }\right)-\text{1}}\right)}{(\text{exp}\left(\Phi x^{\tau }\right)-\text{1}{)}^{\text{2}}}dx. \end{array}$$ (13)

Let $z=\frac{\Phi }{\text{exp}\left(\Phi x^{\tau }\right)-\text{1}}$ and$-dz=\frac{\Phi^{\text{2}}\tau x^{\tau -\text{1}}\text{exp}\left(\Phi x^{\tau }\right)}{(\text{exp}\left(\Phi x^{\tau }\right)-\text{1}{)}^{\text{2}}}$, substituting these two expressions in (13), which take the form


$$\begin{array}{c} =\int_{\text{0}}^{\infty }-x^{r}\text{exp}(-z)dz, \end{array}$$ (14)

Where $x={\left[\frac{log(\Phi /z+\text{1})}{\Phi }\right]}^{\frac{\text{1}}{\tau }}$, then Equation (14) becomes.


$$\begin{array}{c} ={\left(\frac{\text{1}}{\Phi }\right)}^{\frac{\text{1}}{\tau }}\int_{\text{0}}^{\infty }{\left[log(\Phi /\text{2}+\text{1})\right]}^{\frac{\text{1}}{\tau }}\text{exp}(-z)dz, \end{array}$$

For further simplification we used the series representation of


$$\begin{array}{c} {\left[log(\Phi /\text{2}+\text{1})\right]}^{\frac{\text{1}}{\tau }}={\left(-\sum_{k=\text{1}}^{\infty }\frac{(-\text{1}{)}^{k}(\Phi /z{)}^{k}}{k}\right)}^{\frac{\text{1}}{\tau }}. \end{array}$$

Finally, we obtained the result as


$$\begin{array}{c} \mu_{r}=-{\left(-\sum_{k=\text{1}}^{\infty }\frac{(-\text{1}{)}^{k}}{\Phi k}\right)}^{\frac{\text{1}}{\tau }}\Phi^{\frac{k}{\tau }}\Gamma \left((-k-\tau \;)/\;\tau )\;,Z)\;\right). \end{array}$$

It was assuming that τ(k−2τ)≠0,τ(k−τ)≠0.

### 3.4. Order statistics

Let $X_{\text{1}},X_{\text{2}},X_{\text{3}},\ldots .X_{n}$be ordered random variables from ETW, then the PDF of the $i_{th}$ order statistic is given by:


$$\begin{array}{c} f_{x(i)}(x)=\frac{n!}{(i-\text{1})!(n-i)!}F(x{)}^{i-\text{1}}{\left[\text{1}-F(x)\right]}^{n-i}f(x), \end{array}$$ (15)

Using Equation (3) and (4), Equation (15) takes the form:


$$\begin{array}{c} f_{x(i)}(x)=\frac{n!}{(i-\text{1})!(n-i)!}{\left({\text{exp}(}^{-\frac{a}{\text{exp}\left(\Phi x^{\tau }\right)-\text{1}}}\right)}^{i-\text{1}}{\left(\text{1}-\text{exp}\left(-\frac{\Phi }{\text{exp}\left(\Phi x^{\tau }\right)-\text{1}}\right)\right)}^{n-i}\frac{\Phi^{\text{2}}\tau x^{\tau -\text{1}}\text{exp}\left(\Phi x^{\tau }-\frac{\Phi }{{\text{exp}(}^{\Phi x^{\tau }}-\text{1}}\right)}{{\left(\text{exp}\left(\Phi x^{\tau }\right)-\text{1}\right)}^{\text{2}}}, \end{array}$$

The pdf of smallest order statistic of the ETW distribution is given as:


$$\begin{array}{c} f_{x(\text{1})}(x)=\frac{n\Phi^{\text{2}}\tau x^{\tau -\text{1}}\text{exp}\left(\Phi x^{\tau }-\frac{\Phi }{\text{exp}\left(\Phi x^{\tau }\right)-\text{1}}\right)}{{\left(\text{exp}\left(\Phi x^{\tau }\right)-\text{1}\right)}^{\text{2}}}{\left(\text{1}-\text{exp}\left(-\frac{\Phi }{\text{exp}\left(\Phi x^{\tau }\right)-\text{1}}\right)\right)}^{n-\text{1}}, \end{array}$$

The pdf of largest order statistic of the ETW distribution is given as:


$$\begin{array}{c} f_{x(n)}(x)=\frac{n\Phi^{\text{2}}\tau x^{\tau -\text{1}}\text{exp}\left(\Phi x^{\tau }-\frac{\Phi }{\text{exp}\left(\Phi x^{\tau }\right)-\text{1}}\right)}{{\left(\text{exp}\left(\Phi x^{v}\right)-\text{1}\right)}^{\text{2}}}{\left(\text{exp}\left(-\frac{\Phi }{\text{exp}\left(\Phi x^{\tau }\right)-\text{1}}\right)\right)}^{n-\text{1}}. \end{array}$$

### 3.5. Quantile function and median

The quantile function is defined as, $F_{ETW}(x)=u$ where, u(0,1) is uniform random number and $F_{ETW}(x)$ is the CDF of the proposed distribution in (3) which can be solved for a random variable X. The quantile function is defined as


$$\begin{array}{c} \text{exp}\left(\Phi \left(\text{1}-\frac{\text{1}}{\text{1}-\text{exp}\left(-\Phi x^{\tau }\right)}\right)\right)=u, \end{array}$$ (16)

Taking log on both sides of the above expression, we obtain:


$$\begin{array}{c} \Phi \left(\text{1}-\frac{\text{1}}{\text{1}-\text{exp}\left(-\Phi x^{\tau }\right)}\right)=log(u), \end{array}$$

After solving the above expression for x, we obtain: the quantile function as.


$$\begin{array}{c} x={\left[\frac{log\left(\text{1}-\frac{\Phi }{log(u)}\right)}{\Phi }\right]}^{\frac{\text{1}}{\tau }}, \end{array}$$ (17)

To get median put $u=\frac{\text{1}}{\text{2}}$ in Equation (17), we have


$$\begin{array}{c} Med(x)={\left[\frac{log\left(\text{1}+\frac{\Phi }{log(\text{2})}\right)}{\Phi }\right]}^{\frac{\text{1}}{\tau }}. \end{array}$$

### 3.6. The Skewness and Kurtosis

The Quantiles approach evaluates the distribution’s shape, including skewness and kurtosis. Faton and Ibrahim derived Bowley’s skewness formula [9], and the Kurtosis formula by Moors [10] is given as


$$\begin{array}{c} Sk=\frac{Q\left(\frac{\text{3}}{\text{4}}\right)+Q\left(\frac{\text{1}}{\text{4}}\right)-\text{2}Q\left(\frac{\text{1}}{\text{2}}\right)}{Q\left(\frac{\text{3}}{\text{4}}\right)-Q\left(\frac{\text{1}}{\text{4}}\right)}, \end{array}$$

$$\begin{array}{c} Kr=\frac{Q\left(\frac{\text{7}}{\text{8}}\right)+Q\left(\frac{\text{3}}{\text{8}}\right)-Q\left(\frac{\text{5}}{\text{8}}\right)-Q\left(\frac{\text{1}}{\text{3}}\right)}{Q\left(\frac{\text{6}}{\text{8}}\right)-Q\left(\frac{\text{2}}{\text{8}}\right)}. \end{array}$$

Table 1 presents the skewness and kurtosis values of the ETW distribution across various parameter settings.

**Table 1:** Skewness and Kurtosis.

Parameters	Skewness	Kurtosis
a=0.2,b=0.5	0.645918	2.834901
a=0.3,b=0.7	0.483295	1.905418
a=1.2,b=1.8	0.148872	1.248325
a=2.2,b=3.1	0.071233	1.217026
a=3.8,b=4.2	0.044875	1.219259
a=4.9,b=5.3	0.034914	1.221272

### 3.7. Mode

We can get the mode of ETW by taking the derivative of Equation (4) and equating it to zero, then solving for x


$$\begin{array}{c} f(x{)}^{\prime }=\text{0} \end{array}$$

$$\begin{array}{c} \frac{d}{dx}\left(\frac{\Phi^{\text{2}}\tau x^{\tau -\text{1}}\text{exp}\left(\Phi x^{\tau }-\frac{\Phi }{\text{exp}\left(\Phi x^{\tau }\right)-\text{1}}\right)}{{\left(\text{exp}\left(\Phi x^{\tau }\right)-\text{1}\right)}^{\text{2}}}\right)=\text{0}, \end{array}$$

$$\begin{array}{c} -\frac{\Phi^{\tau x^{\tau -\text{2}}}\left[\begin{array}{c} \left(\Phi \tau x^{b}-\tau +\text{1}\right)\text{exp}\left(\text{2}\Phi x^{\tau }\right)+\\ \left(-\Phi^{\text{2}}\tau x^{\tau }+\text{2}\tau -\text{2}\right)\text{exp}\left(\Phi x^{\tau }\right)-\Phi \tau x^{\tau }-\tau +\text{1} \end{array}\right]\text{exp}\left(\Phi x^{\tau }-\frac{\Phi }{{\text{exp}(}^{\Phi x^{\tau }}-\text{1}}\right)}{{\left(\text{exp}\left(\Phi x^{\tau }\right)-\text{1}\right)}^{\text{4}}}=\text{0}, \end{array}$$

$$\begin{array}{c} \left(\Phi \tau x^{\tau }-\tau +\text{1}\right)\text{exp}\left(\text{2}\Phi x^{\tau }\right)+\left(-\Phi^{\text{2}}\tau x^{\tau }+\text{2}\tau -\text{2}\right)\text{exp}\left(\Phi x^{\tau }\right)-\Phi \tau x^{\tau }-\tau +\text{1}=\text{0}. \end{array}$$ (18)

In general, there is not an explicit solution for (18). However, a numerical solution can be obtained by using an iterative procedure.

## 4. Maximum Likelihood Estimation (MLE)

Parameter estimation is conducted using the method of maximum likelihood. Let ${\text{X}}_{\text{1}},{\text{X}}_{\text{2}},\ldots ,{\text{X}}_{\text{n}}$ denote an independent random sample of size n drawn from the ETW distribution. The likelihood function is constructed accordingly, and its logarithm is maximized to obtain the parameter estimates. The likelihood function L can be expressed as follows.


$$\begin{array}{c} L=\prod_{i=\text{1}}^{n}f\left(x_{i};\Phi ,\tau \right), \end{array}$$

Putting (4) in above expression, we obtain:


$$\begin{array}{c} L=\prod_{i=\text{1}}^{n}\frac{\Phi^{\text{2}}\tau x^{\tau -\text{1}}\text{exp}\left(\Phi x^{\tau }-\frac{\Phi }{\text{exp}\left(\Phi x^{\tau }\right)-\text{1}}\right)}{{\left(\text{exp}\left(\Phi x^{\tau }\right)-\text{1}\right)}^{\text{2}}}. \end{array}$$

The log-likelihood function (𝓁) can be obtained by taking the log of expression above.


$$\begin{array}{c} \text{𝓁}=\text{2}n\;log\;\Phi +nlog\tau +(\tau -\text{1})log\sum_{i=\text{1}}^{n}x_{i}+\Phi \sum_{i=\text{1}}^{n}x_{i}^{\tau }-\frac{\Phi }{\text{exp}\left(\Phi \sum_{i=\text{1}}^{n}x_{i}^{\tau }\right)-\text{1}}-\text{2}log\sum_{i=\text{1}}^{n}\left(\text{exp}\left(\Phi {x_{i}}^{\tau }\right)-\text{1}\right), \end{array}$$

The unknown parameters can be determined by taking partial derivatives of 𝓁 with respect to the parameters Φ and τ, setting the results to zero.


$$\begin{array}{c} \frac{\partial \text{𝓁}}{\partial \Phi }=\frac{\text{2}n}{\Phi }+\frac{\Phi \sum_{i=\text{1}}^{n}x_{i}^{\tau }\text{exp}\left(\Phi \sum_{i=\text{1}}^{n}x_{i}^{\tau }\right)}{{\left(\text{exp}\left(\Phi \sum_{i=\text{1}}^{n}x_{i}^{\tau }\right)-\text{1}\right)}^{\text{2}}}-\frac{\text{1}}{\text{exp}\left(\Phi \sum_{i=\text{1}}^{n}x_{i}^{\tau }\right)-\text{1}}+\sum_{i=\text{1}}^{n}x_{i}^{\tau }+\sum_{i=\text{1}}^{n}\frac{\text{2}x_{i}^{\tau }\text{exp}\left(\Phi x_{i}^{\tau }\right)}{\text{exp}\left(\Phi x_{i}^{\tau }\right)-\text{1}}=\text{0}, \end{array}$$ (19)

$$\begin{array}{c} \frac{\partial \text{𝓁}}{\partial \tau }=\frac{n}{\tau }+log\sum_{i=\text{1}}^{n}x_{i}+\frac{\Phi^{\text{2}}\sum_{i=\text{1}}^{n}x_{i}^{\tau }log\sum_{i=\text{1}}^{n}x_{i}\text{exp}\left(\Phi \sum_{i=\text{1}}^{n}x_{i}^{\tau }\right)}{{\left(\text{exp}\left(\Phi \sum_{i=\text{1}}^{n}x_{i}^{\tau }\right)-\text{1}\right)}^{\text{2}}}+\Phi \sum_{i=\text{1}}^{n}x_{i}^{\tau }log\sum_{i=\text{1}}^{n}x_{i}-\sum_{i=\text{1}}^{n}\frac{\text{2}\Phi x_{i}^{\tau }{logx}_{i}\text{exp}\left(\Phi x_{i}^{\tau }\right)}{\text{exp}\left(\Phi x_{i}^{\tau }\right)-\text{1}}=\text{0}. \end{array}$$ (20)

The resulting likelihood Equations (19) and (20) do not admit closed-form solutions; therefore, numerical optimization techniques are required. Alternatively, numerical techniques like the Newton-Raphson and Bisection methods can also be used to obtain the MLEs. In this study, estimation was performed using the optim function in R with the Nelder-Mead algorithm, combined with a multi-start strategy to ensure convergence to the global maximum. The profile log-likelihood functions for the COVID-19 data application are bounded and exhibit a well-defined maximum, confirming the stability of the estimates.

### 4.1. Asymptotic confidence bounds

As previously stated, the unknown parameters do not possess closed-form solutions, precluding closed-form derivation of the exact sampling distribution of the estimators. However, utilizing the asymptotic distribution of MLEs, we have established asymptotic confidence bounds for these unknown values.

The second partial derivatives of the Equation (19) and (20) are respectively given as


$$\begin{array}{cc} I_{\text{1}\text{1}}= & \frac{\partial \text{𝓁}}{\partial \Phi^{\text{2}}}=-\left[\frac{\sum_{i=\text{1}}^{n}x_{i}^{\tau }\text{exp}\left(\Phi \sum_{i=\text{1}}^{n}x_{i}^{\tau }\right)\left\{\left(\text{2}n\sum_{i=\text{1}}^{n}x_{i}^{\tau }-\text{1}\right)\text{exp}\left(\Phi \sum_{i=\text{1}}^{n}x_{i}^{\tau }\right)+\text{2}n\sum_{i=\text{1}}^{n}x_{i}^{\tau }+\text{1}\right\}}{{\left(\text{exp}\left(\Phi \sum_{i=\text{1}}^{n}x_{i}^{\tau }\right)-\text{1}\right)}^{\text{3}}}\right]\\ +\sum_{i=\text{1}}^{n}\frac{\text{2}x_{i}^{\text{2}\tau }\text{exp}\left(\Phi x_{i}^{\tau }\right)}{{\left(\text{exp}\left(\Phi \sum_{i=\text{1}}^{n}x_{i}^{\tau }\right)-\text{1}\right)}^{\text{2}}}, \end{array}$$

$$\begin{array}{cc} I_{\text{2}\text{2}}=\frac{\partial \text{𝓁}}{\partial \tau^{\text{2}}}=\\ & \frac{\Phi \sum_{i=\text{1}}^{n}x_{i}^{\tau }log\sum_{i=\text{1}}^{n}x_{i}^{\text{2}}\left[\text{exp}\left(\Phi \sum_{i=\text{1}}^{n}x_{i}^{\tau }\right)\left(\begin{array}{c} \text{exp}\left(\text{2}\Phi \sum_{i=\text{1}}^{n}x_{i}^{\tau }\right)\\ -\left(\Phi \left(\Phi \sum_{i=\text{1}}^{n}x_{i}^{\tau }-\text{1}\right)+\text{3}\right)\text{exp}\left(\Phi \sum_{i=\text{1}}^{n}x_{i}^{\tau }\right)\\ -\Phi \left(\Phi \sum_{i=\text{1}}^{n}x_{i}^{\tau }+\text{1}\right)+\text{3} \end{array}\right)-\text{1}\right]}{{\left(\text{exp}\left(\Phi \sum_{i=\text{1}}^{n}x_{i}^{\tau }\right)-\text{1}\right)}^{\text{3}}}\\ & -\sum_{i=\text{1}}^{n}\frac{\text{2}\Phi x_{i}^{\tau }{logx}_{i}^{\text{2}}\text{exp}\left(\Phi x_{i}^{\tau }\right)\left(\text{exp}\left(\Phi x_{i}^{\tau }\right)-\Phi x_{i}^{\tau }-\text{1}\right)}{{\left(\text{exp}\left(\Phi x_{i}^{\tau }\right)-\text{1}\right)}^{\text{2}}}, \end{array}$$

$$\begin{array}{cc} I_{\text{1}\text{2}}=\frac{\partial \text{𝓁}}{\partial \Phi \tau }\\ & \;=\frac{\sum_{i=\text{1}}^{n}x_{i}^{\tau }log\sum_{i=\text{1}}^{n}x_{i}\left[\text{exp}\left(\Phi \sum_{i=\text{1}}^{n}x_{i}^{\tau }\right)\left(\begin{array}{c} \text{exp}\left(\text{2}\Phi \sum_{i=\text{1}}^{n}x_{i}^{\tau }\right)-\\ \left(\Phi \left(\Phi \sum_{i=\text{1}}^{n}x_{i}^{\tau }-\text{2}\right)+\text{3}\right)\text{exp}\left(\Phi \sum_{i=\text{1}}^{n}x_{i}^{\tau }\right)-\\ \Phi \left(\Phi \sum_{i=\text{1}}^{n}x_{i}^{\tau }+\text{2}\right)+\text{3} \end{array}\right)-\text{1}\right]}{{\left(\text{exp}\left(\Phi \sum_{i=\text{1}}^{n}x_{i}^{\tau }\right)-\text{1}\right)}^{\text{3}}}\\ & -\sum_{i=\text{1}}^{n}\frac{\text{2}x_{i}^{\tau }{logx}_{i}\text{exp}\left(\Phi x_{i}^{\tau }\right)\left(\text{exp}\left(\Phi x_{i}^{\tau }\right)-\Phi x_{i}^{\tau }-\text{1}\right)}{{\left(\text{exp}\left(\Phi x_{i}^{\tau }\right)-\text{1}\right)}^{\text{2}}}. \end{array}$$

The observed information matrix can be defined as


$$\begin{array}{c} I=-\left(\begin{array}{c} I_{\text{1}\text{1}}I_{\text{1}\text{2}}\\ I_{\text{2}\text{1}}I_{\text{2}\text{2}} \end{array}\right) \end{array}$$

And the variance-covariance matrix is given as


$$\begin{array}{c} V=\left(\begin{array}{c} v_{\text{1}\text{1}}v_{\text{1}\text{2}}\\ v_{\text{2}\text{1}}v_{\text{2}\text{2}} \end{array}\right)={\left(\begin{array}{c} I_{\text{1}\text{1}}I_{\text{1}\text{2}}\\ I_{\text{2}\text{1}}I_{\text{2}\text{2}} \end{array}\right)}^{-\text{1}} \end{array}$$

We used the corresponding MLE to replace the parameters to produce the estimate, which may be defined as


V^=(I11^I12^I21^I22^)−1

We can construct the (1−β)100% confidence intervals using the above variance-covariance matrix for the parameters Φ and τ in the form


Φ∧±Zβ2var(Φ∧),τ^±Zβ2var(τ^)

Where, $Z_{\beta }$ indicates the upper $\beta^{th}$ percentile of standard normal distribution.

## 5. Shannon Entropy

Entropy quantifies the degree of diversity within a given system. One of the famous entropies is Shannon entropy which may be defined as:


$$\begin{array}{c} S_{E}(x)=-\int_{\text{0}}^{\infty }f(x)logf(x)dx, \end{array}$$ (21)

Substituting Equation (4) in (21), we obtain:


$$\begin{array}{c} S_{E}(x)=-\int_{\text{0}}^{\infty }\frac{\Phi^{\text{2}}\tau x^{\tau -\text{1}}\text{exp}\left(\Phi x^{\tau }-\frac{\Phi }{\text{exp}\left(\Phi x^{\tau }\right)-\text{1}}\right)}{{\left(\text{exp}\left(\Phi x^{\tau }\right)-\text{1}\right)}^{\text{2}}}log\left(\frac{\Phi^{\text{2}}\tau x^{\tau -\text{1}}\text{exp}\left(\Phi x^{\tau }-\frac{\Phi }{\text{exp}\left(\Phi x^{\tau }\right)-\text{1}}\right)}{{\left(\text{exp}\left(\Phi x^{\tau }\right)-\text{1}\right)}^{\text{2}}}\right)dx. \end{array}$$ (22)

Equation (22) takes the following form


$$\begin{array}{cc} S_{E}(x)= & -\int_{\text{0}}^{\infty }\frac{\text{2}log\Phi .\Phi^{\text{2}}\tau x^{\tau -\text{1}}\text{exp}\left(\Phi x^{\tau }\right)\text{exp}\left(-\frac{\Phi }{\text{exp}\left(\Phi x^{\tau }\right)-\text{1}}\right)}{{\left(\text{exp}\left(\Phi x^{\tau }\right)-\text{1}\right)}^{\text{2}}}dx\\ & -\int_{\text{0}}^{\infty }\frac{log\tau \Phi^{\text{2}}\tau x^{\tau -\text{1}}\text{exp}\left(\Phi x^{\tau }\right)\text{exp}\left(-\frac{\Phi }{\text{exp}\left(\Phi x^{\tau }\right)-\text{1}}\right)}{{\left(\text{exp}\left(\Phi x^{\tau }\right)-\text{1}\right)}^{\text{2}}}dx\\ & -\int_{\text{0}}^{\infty }\frac{(\tau -\text{1})logx\Phi^{\text{2}}\tau x^{\tau -\text{1}}\text{exp}\left(\Phi x^{\tau }\right)\text{exp}\left(-\frac{\Phi }{\text{exp}\left(\Phi x^{\tau }\right)-\text{1}}\right)}{{\left(\text{exp}\left(\Phi x^{\tau }\right)-\text{1}\right)}^{\text{2}}}dx, \end{array}$$

$$\begin{array}{cc} & -\int_{\text{0}}^{\infty }\frac{\Phi x^{b}\Phi^{\text{2}}\tau x^{\tau -\text{1}}\text{exp}\left(\Phi x^{\tau }\right)\text{exp}\left(-\frac{\Phi }{\text{exp}\left(\Phi x^{\tau }\right)-\text{1}}\right)}{{\left(\text{exp}\left(\Phi x^{\tau }\right)-\text{1}\right)}^{\text{2}}}dx\\ & +\int_{\text{0}}^{\infty }\frac{\Phi^{\text{3}}\tau x^{\tau -\text{1}}\text{exp}\left(\Phi x^{\tau }\right)\text{exp}\left(-\frac{\Phi }{\text{exp}\left(\Phi x^{\tau }\right)-\text{1}}\right)}{{\left(\text{exp}\left(\Phi x^{\tau }\right)-\text{1}\right)}^{\text{4}}}dx\\ & +\int_{\text{0}}^{\infty }\frac{{log\left(\text{exp}\left(\Phi x^{\tau }\right)-\text{1}\right)}^{\text{2}}\Phi^{\text{2}}\tau x^{\tau -\text{1}}\text{exp}\left(\Phi x^{\tau }\right)\text{exp}\left(-\frac{\Phi }{\text{exp}\left(\Phi x^{\tau }\right)-\text{1}}\right)}{{\left(\text{exp}\left(\Phi x^{\tau }\right)-\text{1}\right)}^{\text{2}}}dx, \end{array}$$ (23)

Now, taking the integral of each part in Equation (23) separately, we obtain: the result as:


$$\begin{array}{cc} S_{E}(x)= & -\text{2}\text{log}(\Phi )-\text{log}\tau +\text{log}\Phi \left(\frac{\text{1}+\tau }{\tau }\right)\text{log}\left(\sum \frac{(-\text{1}{)}^{k}}{k}\right)k\left(E_{i}(z)+\text{log}\left(-\frac{\Phi }{z}\right)e^{z}\;\right)|\begin{array}{c} \infty \\ \text{0} \end{array}\;\\ & -\sum \frac{(-\text{1}{)}^{k}}{k}\Phi^{k}(-\text{1}{)}^{\text{2}k}\Gamma (\text{1}-k-z)|\begin{array}{c} \infty \\ \text{0} \end{array}\;-(\text{1}-z)\text{exp}(z)+E_{i}(z)+\text{log}\left(-\frac{\Phi }{z}\right)\text{exp}(z). \end{array}$$

Where $E_{i}$ is the exponential integral. Further, it was assumed that k−2≠0,k−1≠0 and $\sqrt{\left(.\right)}$ is a gamma function.

## 6. Real data application

This section presents an empirical application using COVID-19 survival data to evaluate the practical performance of the proposed model. Additionally, we evaluate the stability of the proposed distribution by comparing it to the Weibull, Transmuted Inverse Weibull (TIW), Inverse Weibull (IW), and Alpha-Power Weibull (APW) models. These models were selected as competitors to provide a tiered comparison: the Weibull as the classic monotonic-hazard model; the IW and TIW as models capable of unimodal hazards; and the APW as a well-regarded, recent three-parameter extension known for its bathtub hazard flexibility.

**Data Set: COVID-19 Patients’ Survival Times in Mexico**

The dataset includes 106 anonymised observations of COVID-19 patients’ period (in days) from diagnosis to death. The information came from documents made public by the Mexican Ministry of Health [29,30]. The values represent individual survival times, not population-level mortality rates. The dataset is as follows:

1.7652, 1.2210, 1.8782, 2.9924, 2.0766, 1.4534, 2.6440, 3.2996, 2.3330, 1.2030, 2.1710, 1.2244,
1.3312, 0.6880, 1.1708, 2.1370, 2.0070, 1.0484, 0.8688, 1.0286, 1.5260, 2.9208, 1.5806, 1.2740,
0.7074, 1.2654, 0.9460, 0.6430, 1.8568, 2.5756, 1.7626, 2.0086, 1.4520, 1.1970, 1.2824, 0.6790,
0.8848, 1.9870, 1.5680, 1.9100, 0.6998, 0.7502, 1.3936, 0.6572, 2.0316, 1.6216, 1.3394, 1.4302,
1.3120, 0.4154, 0.7556, 0.5976, 0.6672, 1.3628, 1.6650, 1.5708, 1.7102, 0.6456, 1.4972, 1.3250,
1.2280, 0.9818, 0.9322, 1.0784, 2.4084, 1.7392, 0.3630, 0.6654, 1.0812, 1.2364, 0.2082, 0.3600,
0.9898, 0.8178, 0.6718, 0.4140, 0.6596, 1.0634, 1.0884, 0.9114, 0.8584, 0.5000, 1.3070, 0.9296,
0.9394, 1.0918, 0.8240, 0.7844, 0.6438, 0.2804, 0.4876, 0.6514, 0.7264, 0.6466, 0.6054, 0.4704,
0.2410, 0.6436, 0.5852, 0.5202, 0.4130, 0.6058, 0.4116, 0.4652, 0.5012 and 0.3846.

**Fig 4:**
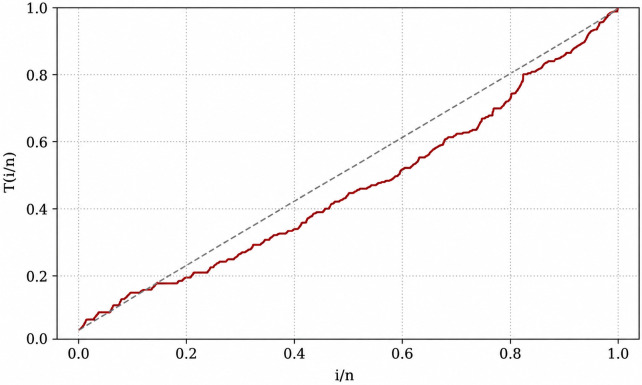
TTT plot of COVID-19 Data.

**Fig 5:**
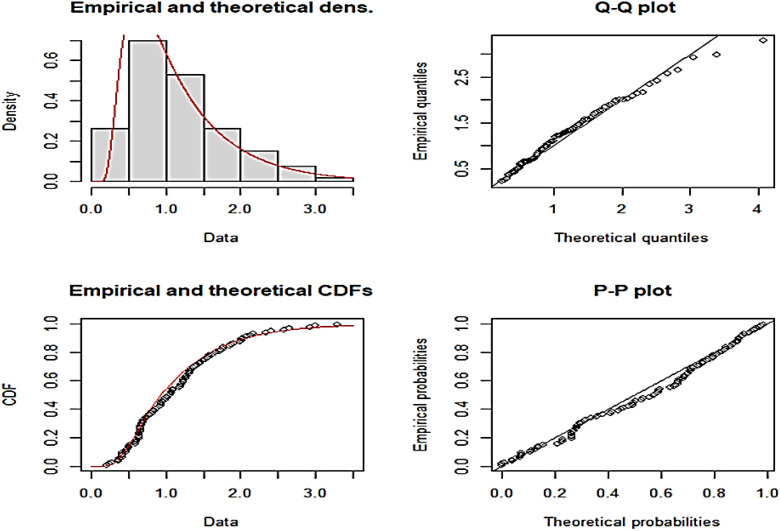
Theoretical, Empirical, QQ & PP Plots of COVID-19 Data.

Fig 4 presents the Total Time on Test (TTT) plot, which provides insight into the underlying hazard rate structure, while Fig 5 demonstrates the theoretical and empirical P-P and Q-Q plots for data set confirming the best fit of the data on ETW distribution. The Total Time on Test (TTT) plot is a graphical tool to identify the shape of the hazard function. It is plotted by calculating

$G\left(\text{r}/\text{n}\right)=[\sum_{i=\text{1}}^{r}T_{i}+(n-r)T_{r}]\;$ for r=1,…,n where T(i) are the ordered survival times. A convex (concave) curve suggests a decreasing (increasing) hazard, while an S-shaped curve, as observed here, suggests a non-monotonic, bathtub-shaped hazard.

Table 2 depicts the MLEs for unknown parameters of ETW distribution. Likewise, Table 3 offers the results of various criteria intended for selecting statistical models. The results of the goodness-of-fit test, shown in Table 3, indicate that the ETW provides a better fit than other well-known distributions.

**Table 2:** Maximum Likelihood Estimates.

Model	Estimates
**ETW**	0.8946919, 1.2616699
**Weibull**	0.6059061, 1.9205049
**Transmuted Inverse Weibull**	1.340692, 1.234306
**Inverse Weibull**	1.1682595, 0.8635808
**Alpha-Power Weibull**	0.2411186, 2.1283381, 0.3892093

**Table 3:** Goodness of fit criteria.

Model	AIC	CAIC	BIC	HQIC
**ETW**	189.7951	189.9116	195.123	191.9541
**Weibull**	192.3865	192.503	197.7134	194.5455
**Transmuted Inverse Weibull**	200.9303	201.0469	206.2572	203.0894
**Inverse Weibull**	246.9029	247.0194	252.2298	249.0619
**Alpha-Power Weibull**	192.304	192.5393	200.2943	195.5425

## 7. Simulation study

This section employs the quantile function (Equation 17) to generate synthetic data from the ETW distribution. 1,000 replications of Monte Carlo simulations are run with different sample sizes and parameter configurations. R (version 4.3.2, R Foundation for Statistical Computing, Vienna, Austria) was used for all statistical analyses. The Nelder-Mead algorithm’s built-in optim() function was used for parameter estimation, along with a multi-start method to guarantee convergence resilience. Standard R procedures and auxiliary packages like numDer were used to calculate numerical derivatives and Hessian evaluations.iv. ggplot2 was used to create diagnostic plots and data visualization. The findings support the consistency and stability of the suggested estimators by showing that bias and mean squared error both decrease with sample size. Mathematically, MSE and Bias are defined as.


$$\begin{array}{c} MSE=\frac{\text{1}}{K}\sum_{i=\text{1}}^{K}{\left({\hat{\theta }}^{i}-\theta \right)}^{\text{2}}, \end{array}$$

$$\begin{array}{c} Bias=\frac{\text{1}}{K}\sum_{i=\text{1}}^{K}\left({\hat{\theta }}^{i}-\theta \right). \end{array}$$

where K=1000 is the number of Monte Carlo replications, ${\hat{\theta }}_{i}$ is the estimate ($\hat{\Phi }$ or $\hat{\tau }$) from the i−th sample, and θ is the true parameter value (Φ or τ). Table 4 summarizes the bias and mean squared error (MSE) of the maximum likelihood estimators for different sample sizes and parameter configurations under the ETW distribution.

**Table 4:** MSE and Bias of ETW Distribution.

a	b	n	MSE(Φ)	Bias(Φ)	MSE(τ)	Bias(τ)
0.02	0.2	30	0.019926	0.071749	0.000881	0.001775
		60	0.004828	0.034237	0.000415	0.000822
		90	0.002803	0.025201	0.000273	0.000767
		120	0.001714	0.019572	0.000202	0.000428
		100	0.002406	0.024206	0.000271	0.000153
		200	0.000669	0.011555	0.000125	0.000668
		300	0.000502	0.009094	8.90E-05	0.0007
0.3	0.03	30	0.043341	0.040909	2.82E-05	0.001533
		60	0.024179	0.013737	1.43E-05	0.001012
		90	0.016235	0.012569	8.46E-06	0.000648
		100	0.01449	0.011915	7.44E-06	0.000453
		200	0.006738	0.007297	3.56E-06	0.000268
		300	0.00503	0.005076	2.40E-06	0.000142
0.2	0.02	30	0.038175	0.058581	1.25E-05	0.001009
		60	0.016242	0.026375	5.96E-06	0.000523
		90	0.011133	0.015124	3.77E-06	0.000384
		100	0.010623	0.019473	3.49E-06	0.000237
		200	0.005172	0.008484	1.73E-06	0.000216
		300	0.003298	0.006335	1.12E-06	0.000119

## 8. Discussion and conclusion

In order to analyze complex lifetime data with non-monotonic hazard rates, this paper establishes the Exponential-Type Weibull (ETW) distribution as a concise yet powerful model. The two-parameter ETW distribution, which is derived from the novel Flexible Exponential-Type Family (FETF) [31], easily accommodates bathtub-shaped and unimodal hazard structures, overcoming important limitations of the classical Weibull model. This capability has been rigorously validated through both theoretical proofs and empirical applications. For the main features of the model, such as the probability density, cumulative distribution, survival, and hazard functions, as well as moments, quantiles, mean residual life, order statistics, and Shannon entropy, we were able to obtain explicit analytical expressions (or, when needed, rapidly convergent series representations).

Furthermore, the maximum likelihood framework was used to systematically build statistical inference and parameter estimation, and the observed Fisher information matrix was used to measure uncertainty. Empirically, likelihood-based measurements, information criteria, and goodness-of-fit diagnostics showed that ETW fit COVID-19 mortality periods from Mexico (n = 106) better than a number of competitors (Weibull, IW, TIW, and APW). Monte Carlo studies with 1,000 repeats showed stable model selection and consistent parameter recovery (decreasing MSE and bias with increasing sample size).

The ETW is appealing in applied contexts where over-parameterization is a concern or sample sizes are small because of its two-parameter parameter parsimony and hazard flexibility. Potential extensions include (i) adapting estimation methods to handle right-censored data (for example, via EM algorithms or partial likelihood approaches), and (ii) embedding the ETW within regression frameworks (accelerated failure time or proportional hazards) to accommodate covariate effects.

In conclusion, the ETW distribution offers a useful and comprehensible tool for modeling complicated lifetime data by striking an efficient compromise between flexibility and parsimony. It is a useful contribution to the class of contemporary survival models due to its strong theoretical characteristics and proven empirical performance.

## Supporting information

S1 File R Scripts for Visualization and statistical analysis. (PDF)

S2 File Figs 2 and 3 (update R codes). (DOCX)
