# Supplementary material for: A flexible exponential type family for modeling non-monotonic hazard rates with application to mortality analysis: A COVID-19 case study
Source: PLoS One. 2026 Aug 6;21(8):e0331050. doi: 10.1371/journal.pone.0331050 (PMC13446746; doi:10.1371/journal.pone.0331050)
Supplement: Supplementary file 1 [file pone.0331050.s001.pdf]

1) plot_density_ETW

# plot_density.R

# Purpose: Plot ETW-like density curves for multiple (alpha,beta) parameter combinations.

# Output: density_plot.png

```
x <- seq(0, 5, by = 0.01)

# ETW-like pdf used in manuscript (same algebraic shape as your sample)
dETW <- function(x, a, b) {
  eps <- 1e-12
  ex <- exp(b * x)
  denom <- (ex - 1)^2 + eps
  pdf <- (a * b * exp(b * x - a / (ex - 1 + eps))) / denom
  return(pdf)
}

# parameter sets (in the same order/style as your sample)
params <- list(c(0.3,1.3), c(0.4,1.3), c(0.4,2.1), c(0.01,1.2))
cols <- c("red", "blue", "black", "green")
lts <- c(1,2,3,4)
lws <- rep(3,4)

# compute densities
ylist <- lapply(params, function(p) dETW(x, p[1], p[2]))

# plot -> PNG
png("density_plot.png", width=900, height=600, res=150)
plot(x, ylist[[1]], type="l", col=cols[1], lwd=lws[1], xlab="x", ylab="f(x)",
     main="ETW-like PDF for selected (a,b)")
```

```
lines(x, ylist[[2]], lty=lts[2], col=cols[2], lwd=lws[2])
lines(x, ylist[[3]], lty=lts[3], col=cols[3], lwd=lws[3])
lines(x, ylist[[4]], lty=lts[4], col=cols[4], lwd=lws[4])
legend("topright",
       legend = c(expression(paste(alpha, " = ", "0.3, ", beta, "=",1.3)),
                  expression(paste(alpha, " = ", "0.4, ", beta, "=",1.3)),
                  expression(paste(alpha, " = ", "0.4, ", beta, "=",2.1)),
                  expression(paste(alpha, " = ", "0.01, ", beta, "=",1.2))),
       lty = lts, col = cols, lwd = lws, cex = 0.8)
dev.off()
cat("Saved density_plot.png\n")
```

2) plot_pdf_cdf.R {PDF and CDF strip (Figure 2)}

```
# plot_pdf_cdf.R
# Purpose: Plot ETW PDF and CDF for a selection of parameters.
# Output: etw_pdf.png, etw_cdf.png

# ETW CDF and PDF
pETW <- function(x, a, b) {
  ex <- exp(b * x)
  cdf <- 1 - exp(- a / (ex - 1 + 1e-12))
  return(cdf)
}
dETW <- function(x, a, b) {
  ex <- exp(b * x)
  denom <- (ex - 1)^2 + 1e-12
  pdf <- (a * b * ex * exp(- a/(ex - 1 + 1e-12))) / denom
  return(pdf)
}
```

```
x <- seq(0.01, 5, by = 0.01)
params <- list(c(0.2,1.3), c(0.3,1.8), c(0.4,0.9), c(0.5,1.2))
cols <- c("red","blue","black","green")

png("etw_pdf.png", width=900, height=600, res=150)
plot(x, dETW(x, params[[1]][1], params[[1]][2]), type="l", lwd=3, col=cols[1],
   xlab="x", ylab="f(x)", main="ETW PDF")
for(i in 2:length(params)) lines(x, dETW(x, params[[i]][1], params[[i]][2]), col=cols[i], lwd=3,
lty=i)
legend("topright", legend = sapply(params, function(p) paste0("a=",p[1],", b=",p[2])),
     col=cols, lwd=3, lty=1:length(params), cex=0.8)
dev.off()

png("etw_cdf.png", width=900, height=600, res=150)
plot(x, pETW(x, params[[1]][1], params[[1]][2]), type="l", lwd=3, col=cols[1],
   xlab="x", ylab="F(x)", main="ETW CDF")
for(i in 2:length(params)) lines(x, pETW(x, params[[i]][1], params[[i]][2]), col=cols[i], lwd=3,
lty=i)
legend("bottomright", legend = sapply(params, function(p) paste0("a=",p[1],", b=",p[2])),
     col=cols, lwd=3, lty=1:length(params), cex=0.8)
dev.off()
cat("Saved etw_pdf.png and etw_cdf.png\n")
```

3) plot_hazard.R {hazard strip (Figure 3)}

```
# plot_hazard.R
# Purpose: Plot hazard function of ETW for several parameter values.
# Output: etw_hazard.png
```

```
dETW <- function(x, a, b) {
  ex <- exp(b * x)
  denom <- (ex - 1)^2 + 1e-12
  pdf <- (a * b * ex * exp(- a/(ex - 1 + 1e-12))) / denom
  return(pdf)
}
pETW <- function(x, a, b) {
  ex <- exp(b * x)
  cdf <- 1 - exp(- a / (ex - 1 + 1e-12))
  return(cdf)
}
hETW <- function(x, a, b) {
  pdf <- dETW(x, a, b)
  surv <- 1 - pETW(x, a, b)
  return(pdf / (surv + 1e-12))
}

x <- seq(0.01, 5, by=0.01)
params <- list(c(0.5,0.8), c(2.5,2.5), c(0.2,1.3))
cols <- c("red","blue","black")

png("etw_hazard.png", width=900, height=600, res=150)
plot(x, hETW(x, params[[1]][1], params[[1]][2]), type="l", lwd=3, col=cols[1],
     xlab="x", ylab="h(x)", main="ETW Hazard Function")
for(i in 2:length(params)) lines(x, hETW(x, params[[i]][1], params[[i]][2]), col=cols[i], lwd=3,
lty=i)
legend("topright", legend = sapply(params, function(p) paste0("a=",p[1],", b=",p[2])),
       col=cols, lwd=3, lty=1:length(params))
dev.off()
cat("Saved etw_hazard.png\n")
```

4) plot_ttt.R:{Total Time on Test (TTT) plot (Figure 4)}

```
# plot_ttt.R
# Purpose: Compute and plot the Total Time on Test (TTT) plot for the COVID-19 data
# Output: ttt_plot.png

x <- c(1.7652,1.2210,1.8782,2.9924,2.0766,1.4534,2.6440,3.2996,2.3330,1.2030,2.1710,1.2244,
1.3312,0.6880,1.1708,2.1370,2.0070,1.0484,0.8688,1.0286,1.5260,2.9208,1.5806,1.2740,
0.7074,1.2654,0.9460,0.6430,1.8568,2.5756,1.7626,2.0086,1.4520,1.1970,1.2824,0.6790,
0.8848,1.9870,1.5680,1.9100,0.6998,0.7502,1.3936,0.6572,2.0316,1.6216,1.3394,1.4302,
1.3120,0.4154,0.7556,0.5976,0.6672,1.3628,1.6650,1.5708,1.7102,0.6456,1.4972,1.3250,
1.2280,0.9818,0.9322,1.0784,2.4084,1.7392,0.3630,0.6654,1.0812,1.2364,0.2082,0.3600,
0.9898,0.8178,0.6718,0.4140,0.6596,1.0634,1.0884,0.9114,0.8584,0.5000,1.3070,0.9296,
0.9394,1.0918,0.8240,0.7844,0.6438,0.2804,0.4876,0.6514,0.7264,0.6466,0.6054,0.4704,
0.2410,0.6436,0.5852,0.5202,0.4130,0.6058,0.4116,0.4652,0.5012,0.3846)

n <- length(x)
x_ord <- sort(x)
x_bar <- mean(x)
TTT <- sapply(1:n, function(r) (sum(x_ord[1:r]) + (n - r) * x_ord[r]) / (n * x_bar))

png("ttt_plot.png", width=900, height=600, res=150)
plot((1:n)/n, TTT, type='b', xlab='r/n', ylab='TTT(r/n)', main='Total Time on Test (TTT) Plot')
abline(a=0, b=1, col="gray")
dev.off()
cat("Saved ttt_plot.png\n")
```

5) qq_pp_plots.R:{QQ & PP plots (Figure 5)}

```
# qq_pp_plots.R
```

```r
# Purpose: Generate theoretical QQ and PP plots for ETW fitted to COVID-19 data.
# Output: qq_plot.png, pp_plot.png

# Data
x <- c(1.7652,1.2210,1.8782,2.9924,2.0766,1.4534,2.6440,3.2996,2.3330,1.2030,2.1710,1.2244,
1.3312,0.6880,1.1708,2.1370,2.0070,1.0484,0.8688,1.0286,1.5260,2.9208,1.5806,1.2740,
0.7074,1.2654,0.9460,0.6430,1.8568,2.5756,1.7626,2.0086,1.4520,1.1970,1.2824,0.6790,
0.8848,1.9870,1.5680,1.9100,0.6998,0.7502,1.3936,0.6572,2.0316,1.6216,1.3394,1.4302,
1.3120,0.4154,0.7556,0.5976,0.6672,1.3628,1.6650,1.5708,1.7102,0.6456,1.4972,1.3250,
1.2280,0.9818,0.9322,1.0784,2.4084,1.7392,0.3630,0.6654,1.0812,1.2364,0.2082,0.3600,
0.9898,0.8178,0.6718,0.4140,0.6596,1.0634,1.0884,0.9114,0.8584,0.5000,1.3070,0.9296,
0.9394,1.0918,0.8240,0.7844,0.6438,0.2804,0.4876,0.6514,0.7264,0.6466,0.6054,0.4704,
0.2410,0.6436,0.5852,0.5202,0.4130,0.6058,0.4116,0.4652,0.5012,0.3846)

# ETW MLE estimates
a_hat <- 0.8946919
b_hat <- 1.2616699

pETW <- function(x, a, b) {
  ex <- exp(b * x)
  cdf <- 1 - exp(- a / (ex - 1 + 1e-12))
  return(cdf)
}

# QQ-plot using numerical inversion of CDF
n <- length(x)
pvals <- (1:n) / (n + 1)
theo_q <- sapply(pvals, function(p) {
  uniroot(function(t) pETW(t, a_hat, b_hat) - p, lower=1e-6, upper=50)$root
})
```

```
png("qq_plot.png", width=900, height=600, res=150)
plot(sort(x), theo_q, xlab="Sample Quantiles", ylab="Theoretical Quantiles (ETW)", main="QQ
Plot: ETW vs Data")
abline(0,1, col="gray")
dev.off()

# PP-plot
emp_cdf <- ecdf(x)
model_cdf_vals <- sapply(sort(x), function(xi) pETW(xi, a_hat, b_hat))
png("pp_plot.png", width=900, height=600, res=150)
plot(emp_cdf(sort(x)), model_cdf_vals, xlab="Empirical CDF", ylab="Model CDF (ETW)",
main="PP Plot: ETW vs Data")
abline(0,1, col="gray")
dev.off()
cat("Saved qq_plot.png and pp_plot.png\n")
```

6) likelihood_plots.R: likelihood surface + profile likelihoods

```
# likelihood_plots.R
# Purpose: Compute and plot log-likelihood surface and profile likelihoods for ETW.
# Output: ll_surface.png, profile_likelihoods.png

x <- c(1.7652,1.2210,1.8782,2.9924,2.0766,1.4534,2.6440,3.2996,2.3330,1.2030,2.1710,1.2244,
1.3312,0.6880,1.1708,2.1370,2.0070,1.0484,0.8688,1.0286,1.5260,2.9208,1.5806,1.2740,
0.7074,1.2654,0.9460,0.6430,1.8568,2.5756,1.7626,2.0086,1.4520,1.1970,1.2824,0.6790,
0.8848,1.9870,1.5680,1.9100,0.6998,0.7502,1.3936,0.6572,2.0316,1.6216,1.3394,1.4302,
1.3120,0.4154,0.7556,0.5976,0.6672,1.3628,1.6650,1.5708,1.7102,0.6456,1.4972,1.3250,
1.2280,0.9818,0.9322,1.0784,2.4084,1.7392,0.3630,0.6654,1.0812,1.2364,0.2082,0.3600,
0.9898,0.8178,0.6718,0.4140,0.6596,1.0634,1.0884,0.9114,0.8584,0.5000,1.3070,0.9296,
0.9394,1.0918,0.8240,0.7844,0.6438,0.2804,0.4876,0.6514,0.7264,0.6466,0.6054,0.4704,
```

```
0.2410,0.6436,0.5852,0.5202,0.4130,0.6058,0.4116,0.4652,0.5012,0.3846)

loglik <- function(par, data) {
  a <- par[1]; b <- par[2]
  ex <- exp(b * data)
  denom <- (ex - 1)^2 + 1e-12
  pdf <- (a * b * ex * exp(- a/(ex - 1 + 1e-12))) / denom
  ll <- sum(log(pdf + 1e-12))
  return(ll)
}

# Grid for surface
a_seq <- seq(0.1, 2.0, length.out = 80)
b_seq <- seq(0.2, 3.0, length.out = 80)
LL <- matrix(NA, nrow = length(a_seq), ncol = length(b_seq))
for(i in seq_along(a_seq)){
  for(j in seq_along(b_seq)){
    LL[i,j] <- loglik(c(a_seq[i], b_seq[j]), x)
  }
}

# contour
png("ll_surface.png", width=900, height=700, res=150)
contour(a_seq, b_seq, t(LL), xlab="a", ylab="b", main="Log-likelihood surface (ETW)")
dev.off()

# profile likelihoods (maximize over the other parameter)
profile_a <- apply(LL, 1, max, na.rm=TRUE)
profile_b <- apply(LL, 2, max, na.rm=TRUE)
a_mle <- a_seq[which.max(profile_a)]
b_mle <- b_seq[which.max(profile_b)]
```

```
png("profile_likelihoods.png", width=900, height=600, res=150)
par(mfrow=c(1,2))
plot(a_seq, profile_a, type='l', xlab="a", ylab="Profile log-lik",
     main=paste("Profile Likelihood for a (MLE a=",round(a_mle,3),")"))
abline(v=a_mle, col="red", lty=2)
plot(b_seq, profile_b, type='l', xlab="b", ylab="Profile log-lik",
     main=paste("Profile Likelihood for b (MLE b=",round(b_mle,3),")"))
abline(v=b_mle, col="red", lty=2)
dev.off()
cat("Saved ll_surface.png and profile_likelihoods.png\n")
```

7) simulation_study.R: Monte Carlo replicates for Table 4

```
# simulation_study.R
# Purpose: Monte Carlo study that generates ETW samples and fits MLE, producing bias and MSE estimates.
# Output: simulation_results.csv and printed bias/MSE

set.seed(123)

# ETW CDF/PDF/quantile via numerical inversion
pETW <- function(x, a, b) {
  ex <- exp(b * x)
  cdf <- 1 - exp(- a / (ex - 1 + 1e-12))
  return(cdf)
}
dETW <- function(x, a, b) {
  ex <- exp(b * x)
  denom <- (ex - 1)^2 + 1e-12
```

```
  pdf <- (a * b * ex * exp(- a/(ex - 1 + 1e-12))) / denom
  return(pdf)
}
qETW <- function(p, a, b) {
  uniroot(function(t) pETW(t, a, b) - p, lower=1e-8, upper=100)$root
}
rETW <- function(n, a, b) {
  u <- runif(n)
  sapply(u, function(p) qETW(p, a, b))
}

# Negative log-likelihood for optim
nll <- function(par, data) {
  a <- par[1]; b <- par[2]
  pdf <- dETW(data, a, b)
  return(-sum(log(pdf + 1e-12)))
}

# Simulation settings (example block; adjust as needed for Table 4)
R <- 500   # replicates (use 1000 for final runs)
sample_sizes <- c(30, 60, 90, 120, 200, 300)
true_params <- list(c(0.2, 0.02), c(0.3, 0.03), c(0.2, 0.02))  # example columns from Table 4;
replace as needed

results_list <- list()

for(tp in seq_along(true_params)){
  a_true <- true_params[[tp]][1]
  b_true <- true_params[[tp]][2]
  res_table <- data.frame(n=integer(), bias_a=numeric(), bias_b=numeric(), mse_a=numeric(),
mse_b=numeric(), stringsAsFactors=FALSE)
```

```
  for(n in sample_sizes){
    ests <- matrix(NA, nrow=R, ncol=2)
    for(r in 1:R){
      dat <- rETW(n, a_true, b_true)
      # starting values
      start <- c(max(1e-3, a_true), max(1e-3, b_true))
      fit <- try(optim(par=start, fn=nll, data=dat, method="Nelder-Mead",
control=list(maxit=2000)), silent=TRUE)
      if(inherits(fit, "try-error")) ests[r,] <- c(NA,NA) else ests[r,] <- fit$par
    }
    a_hat <- ests[,1]; b_hat <- ests[,2]
    bias_a <- mean(a_hat, na.rm=TRUE) - a_true
    bias_b <- mean(b_hat, na.rm=TRUE) - b_true
    mse_a <- mean((a_hat - a_true)^2, na.rm=TRUE)
    mse_b <- mean((b_hat - b_true)^2, na.rm=TRUE)
    res_table <- rbind(res_table, data.frame(n=n, bias_a=bias_a, bias_b=bias_b, mse_a=mse_a,
mse_b=mse_b))
  }
  results_list[[paste0("true_",tp)]] <- res_table
  # save each block
  write.csv(res_table, file=paste0("simulation_block_",tp,".csv"), row.names=FALSE)
}
# Collate summary
saveRDS(results_list, file="simulation_summary.rds")
cat("Saved simulation_block_*.csv and simulation_summary.rds\n")
```
