# Supplementary material for: A flexible exponential type family for modeling non-monotonic hazard rates with application to mortality analysis: A COVID-19 case study
Source: PLoS One. 2026 Aug 6;21(8):e0331050. doi: 10.1371/journal.pone.0331050 (PMC13446746; doi:10.1371/journal.pone.0331050)
Supplement: Supplementary file 2 [file pone.0331050.s006.pdf]

Response to Editor’s Additional Comment

Dear Professor Alessandro Barbiero,

Thank you for your valuable comment regarding Figures 2 and 3.

We have carefully revised both figures to ensure that all curves are fully contained within the plotting region. The axis limits have been adjusted appropriately so that no portion of any curve extends outside the frame. The updated figures now clearly display all results without overlap or truncation.

We hope the revised figures now meet the journal’s presentation standards.

Kind regards,

Dr. Bilal Himmat (on behalf of all authors)
