# Supplementary material for: A flexible exponential type family for modeling non-monotonic hazard rates with application to mortality analysis: A COVID-19 case study
Source: PLoS One. 2026 Aug 6;21(8):e0331050. doi: 10.1371/journal.pone.0331050 (PMC13446746; doi:10.1371/journal.pone.0331050)
Supplement: Supplementary file 3 [file pone.0331050.s002.docx]

#=========================================================

\# Figure 2: PDF and CDF of ETW Model

#=========================================================

par(mfrow = c(1, 2), mar = c(4.5, 4.5, 2, 1))

\# Grid

x <- seq(0.001, 1.5, by = 0.01)

#---------------------------

\# PDF of ETW

#---------------------------

pdf_etw <- function(x, a, b) {

(a * b * exp(b * x - a / (exp(b * x) - 1))) /

(exp(b * x) - 1)^2

}

f1 <- pdf_etw(x, 0.3, 1.3)

f2 <- pdf_etw(x, 0.4, 1.3)

f3 <- pdf_etw(x, 0.4, 2.1)

f4 <- pdf_etw(x, 0.1, 1.2)

ylim1 <- max(c(f1, f2, f3, f4))

plot(x, f1,

type = "l",

col = "red",

lwd = 3,

ylim = c(0, ylim1 * 1.05),

xlab = "x",

ylab = "f(x)",

main = "PDF of ETW")

lines(x, f2, lty = 2, col = "blue", lwd = 3)

lines(x, f3, lty = 3, col = "black", lwd = 3)

lines(x, f4, lty = 4, col = "darkgreen", lwd = 3)

legend("topright",

legend = c(

expression(alpha==0.3~","~beta==1.3),

expression(alpha==0.4~","~beta==1.3),

expression(alpha==0.4~","~beta==2.1),

expression(alpha==0.1~","~beta==1.2)

),

lty = 1:4,

col = c("red","blue","black","darkgreen"),

lwd = 3,

bty = "n",

cex = 0.85)

#---------------------------

\# CDF of ETW (numerical approximation)

#---------------------------

cdf_etw <- function(x, a, b) {

dx <- x[2] - x[1]

fx <- pdf_etw(x, a, b)

cumsum(fx) * dx

}

F1 <- cdf_etw(x, 0.3, 1.3)

F2 <- cdf_etw(x, 0.4, 1.3)

F3 <- cdf_etw(x, 0.4, 2.1)

F4 <- cdf_etw(x, 0.1, 1.2)

plot(x, F1,

type = "l",

col = "red",

lwd = 3,

ylim = c(0, 1),

xlab = "x",

ylab = "F(x)",

main = "CDF of ETW")

lines(x, F2, lty = 2, col = "blue", lwd = 3)

lines(x, F3, lty = 3, col = "black", lwd = 3)

lines(x, F4, lty = 4, col = "darkgreen", lwd = 3)

legend("bottomright",

legend = c(

expression(alpha==0.3~","~beta==1.3),

expression(alpha==0.4~","~beta==1.3),

expression(alpha==0.4~","~beta==2.1),

expression(alpha==0.1~","~beta==1.2)

),

lty = 1:4,

col = c("red","blue","black","darkgreen"),

lwd = 3,

bty = "n",

cex = 0.85)

#=========================================================

\# Figure 3: Hazard Function

#=========================================================

par(mar = c(4.5, 4.5, 1.5, 1))

\# Grid (avoid zero for stability)

x <- seq(0.001, 1.5, by = 0.01)

#-----------------------------

\# PDF of ETW

#-----------------------------

pdf_etw <- function(x, a, b) {

(a * b * exp(b * x - a / (exp(b * x) - 1))) /

(exp(b * x) - 1)^2

}

#-----------------------------

\# CDF (numerical integration)

#-----------------------------

cdf_etw <- function(x, a, b) {

dx <- x[2] - x[1]

fx <- pdf_etw(x, a, b)

cumsum(fx) * dx

}

#-----------------------------

\# Hazard function

#-----------------------------

hazard_etw <- function(x, a, b) {

fx <- pdf_etw(x, a, b)

Fx <- cdf_etw(x, a, b)

fx / (1 - Fx)

}

#-----------------------------

\# Parameter sets

#-----------------------------

h1 <- hazard_etw(x, 0.2, 1.3)

h2 <- hazard_etw(x, 0.3, 1.8)

h3 <- hazard_etw(x, 0.4, 0.9)

h4 <- hazard_etw(x, 0.5, 1.2)

#-----------------------------

\# Avoid extreme values (frame control)

#-----------------------------

ylim.max <- quantile(c(h1, h2, h3, h4), 0.98, na.rm = TRUE)

#-----------------------------

\# Plot

#-----------------------------

plot(x, h1,

type = "l",

col = "red",

lwd = 3,

ylim = c(0, ylim.max * 1.05),

xlab = "x",

ylab = "h(x)",

main = "Hazard Function of ETW Model")

lines(x, h2, lty = 2, col = "blue", lwd = 3)

lines(x, h3, lty = 3, col = "black", lwd = 3)

lines(x, h4, lty = 4, col = "darkgreen", lwd = 3)

#-----------------------------

\# Legend (bottom-right as requested earlier style)

#-----------------------------

legend("bottomright",

legend = c(

expression(alpha==0.2~","~beta==1.3),

expression(alpha==0.3~","~beta==1.8),

expression(alpha==0.4~","~beta==0.9),

expression(alpha==0.5~","~beta==1.2)

),

lty = 1:4,

col = c("red","blue","black","darkgreen"),

lwd = 3,

bty = "n",

cex = 0.9)
