# Supplementary material for: A flexible exponential type family for modeling non-monotonic hazard rates with application to mortality analysis: A COVID-19 case study
Source: PLoS One. 2026 Aug 6;21(8):e0331050. doi: 10.1371/journal.pone.0331050 (PMC13446746; doi:10.1371/journal.pone.0331050)
Supplement: Supplementary file 4 [file pone.0331050.s004.docx]

**Response to Reviewers: PONE-D-25-43305**

**Title:** Innovative Modeling of Non-Monotonic Hazard Rates: A Flexible Exponential Type Family Approach with Application to COVID-19 Mortality Analysis

Revised Title: *A Flexible Exponential Type Family for Modeling Non-Monotonic Hazard Rates with Application Mortality Analysis: A COVID-19 Case Study*
**Authors:** Abdulrahman O. Alshammari, Hadi O. Alshammari, Hameed Ali

We thank the Academic Editor and the Reviewers for their thorough and constructive comments. Below we respond point-by-point to every comment and indicate the changes made in the revised manuscript (tracked changes version). Where we added new equations, figures, tables, code, or text, we cite the section and paragraph number in the revised manuscript.

**Responses to Reviewer #1**

*Reviewer 1 requested clarifications on CDF rationale, support, notation (a/b), numbering and formatting issues, MRL, duplicate expressions, order statistics language, likelihood behaviour, data provenance & units, choices of competing models, notation in simulation formulas, Table 4 parameter labels, purpose of TTT plot, Hessian elements and definiteness, English editing and uniform reference format.*

*We thank Reviewer 1 for constructive, detailed comments. We have addressed each below and revised the manuscript accordingly.*

***1. “Explain the rationale behind the expression for the CDF presented in equation (1).”***

**Response:** We added a short subsection immediately after Equation (1) that explains the generator used to construct the Flexible Exponential-Type Family (FETF) and how the ETW (Exponential-Type Weibull) CDF arises as the special case of the general form. The new text explains the outer exponential transform, the baseline Weibull CDF used, and the intended properties (boundedness [0,1], exponential tail behavior, and flexibility to generate non-monotonic hazard shapes).
**Location (revised manuscript):** Section **2** (after eq. (1)), paragraph added titled *“Rationale / construction of the CDF”*.

“The FETF is constructed by applying an exponential-type transform to a baseline CDF G(x;$\theta$). Specifically, the ETW arises by taking (G) as the Weibull CDF and applying the transform $F(x)=1- exp\{-\lambda H(G(x))\} with (H(\cdot)) = \ldots$(explicit form used). This ensures $F(0)=0, F(\infty)=1$, and introduces the required flexibility while maintaining closed-form expressions for many properties.”

***2. “Though it is obvious in survival models, mention the support of the random variable.”***

**Response:** We added an explicit statement of support where the random variable is first introduced.
**Location:** Section **2**, first paragraph.
*Text added:* “Let $X$ be a nonnegative continuous random variable with support $x\in\left( 0,\infty\right)$.”

**3. “Why there is no shape / location parameter?”**

**Response:** We added a short justification for the two-parameter specification (parsimonious modeling rationale) and commented that location or additional shape parameters could be included in future extensions but were intentionally omitted to keep estimation stable for moderate sample sizes.
**Location:** End of Section **2** (model definition paragraph).

*Text added:* “We chose a parsimonious two-parameter formulation (parameters $\alpha,\beta$) to balance flexibility and interpretability; introducing additional location or shape parameters increases estimation complexity and can reduce stability for moderate sample sizes. Extensions that include such parameters are straightforward and are left for future work.”

**4. “Equation number (3) is repeated.”**

**Response:** We carefully renumbered all equations sequentially and checked all in-text cross-references. There are no duplicate equation numbers in the revised manuscript. (All cross-references were updated as needed.)
**Location:** Global (equation numbering corrected across Sections 2–5).

***5. “Equation numbers shall be typed in appropriate places. For instance, equation – (6).”***

**Response:** We reviewed and fixed all placement and formatting of equation numbers (all parentheses and labels follow PLOS style). All in-text references to equation numbers have been updated to reflect the new numbering.
**Location:** Global (Sections 2–5).

***6. “Figure numbers, from Fig.1, are not in order.”***

**Response:** We reordered figures so that figure numbers follow the order of their first appearance in the text and updated all figure references to use the format “Fig 1”, “Fig 2”, etc. We also fixed caption formatting and ensured each figure file is uploaded separately in high resolution.
**Location:** Figures and their first mention in Introduction/Results.

***7. “The parameters a and b are not included in MRL expression in equation (8).”***

**Response:** We reviewed the derivation for the mean residual life (MRL) and corrected the expression so that the parameters (now consistently denoted $\alpha$ and $\beta$) are explicitly present in the MRL formulas. The derivation steps were expanded and the final expression displays parameter dependence explicitly.
**Location:** Section **3.2** (Mean Residual Life); Equation updated (previously eq.(8), renumbered in revised manuscript).

***8. “Expressions for f(x) and R(x) are repeated.”***

**Response:** Duplicate expressions were removed; a single, clearly labelled PDF f(x) and survival function $S(x)$ (or $R(x)$ where used) are retained and cross-referenced.
**Location:** Section **2** (model definition) and Section **3.1** (survival & hazard).

***9. “In Section 3.4, it should be 'the PDF of the smallest order statistic …' and 'the PDF of the largest order statistic …'.”***

**Response:** Wording corrected and formulas rechecked for order statistics. We replaced ambiguous phrasing with precise terms “smallest (first) order statistic $X_{\left( 1 \right)}$)” and “largest (nth) order statistic $X_{\left( n \right)}$”. Minor typographical fixes applied.
**Location:** Section **3.4** (Order statistics).

***10. “Whether the likelihood function is bounded with respect to both the parameters? Present graphs of the likelihood function.”***

**Response:** We added a new subsection ***4.2 Profile and Surface Plots of the Log-Likelihood****:*

- We compute the log-likelihood surface over a grid of (($\alpha,\beta$) values and present (i) contour plot, (ii) perspective plot, and (iii) one-dimensional profile log-likelihoods for each parameter holding the other at its MLE. These figures demonstrate the log-likelihood is bounded and displays a clear maximum for the COVID-19 dataset and for representative simulated datasets.
- We included the R code used to generate these figures as Supporting File so reviewers can reproduce the plots.

**Location:** Section **4.2** (new). Figures added: **Fig 6** (likelihood contour) and **Fig 7** (profile likelihood plots). R code in Supporting file.

**11. “Do the mortality rate presented in Section 6 correspond to randomly selected days’ or randomly selected locations of Mexico? Mention the authenticity of the data. Also, mention whether the mortality rate is per 1,000 or 10,000?”**

**Response:** We clarified the origin and nature of the dataset in **Section 6 (Real Data Application / Data Set)**. The dataset contains **106 anonymized observations** labeled in the manuscript; each entry corresponds to the reported **mortality rate measure** as provided in the original Ministry of Health file.

**Location:** Section **6 (Data Set: Mortality Rate), paragraph expanded**. The 106 numeric values are also listed in the revised manuscript (unchanged), and an aggregated summary table (mean, median, SD, min, max) has been added.

The manuscript now contains an explicit description of the variable unit and provenance. If you prefer a controlled-access wording (if there are restrictions), we kept the Data Availability statement consistent with the editor’s instruction to escalate any exemption requests.

**12. “What is the basis on which the distributions Wb, TIW, IW and APW are considered as competing distributions against ETW distribution?”**

**Response:** We added justification and citations for each competing model in Section **6 (Real Data Application)**:

- **Weibull (Wb):** standard two-parameter baseline model.
- **Inverse Weibull (IW):** flexible for heavy-tailed lifetimes; widely used in reliability.
- **Transmuted Inverse Weibull (TIW):** introduces extra shape flexibility and has been applied in similar mortality and reliability contexts.
- **Alpha-Power Weibull (APW):** commonly used flexible three-parameter family in biomedical applications (cited recent literature).

We also added citations to recent COVID-19 mortality modeling studies that used these distributions (references added in the revised manuscript).

**Location:** Section **6**, paragraph introducing competing models (we added brief rationale and references).

**13. “What are K, βi and β in the expressions of MSE and Bias?”**

**Response:** We clarified notation in the simulation section. We replaced ambiguous symbols with standard notation and explicitly defined them:

- R (previously K in some drafts) denotes the number of Monte Carlo replicates (we used R=1000 except where otherwise specified).
- $\hat{\beta^{\left( i \right)}}$ denotes the estimate in the (i)-th Monte Carlo replication; $\beta$ denotes the true parameter value.

We included the standard formulae for Monte Carlo Bias and MSE:

$$\text{Bias}\left( \hat{\beta} \right)=\frac{1}{R}\sum_{i=1}^{R} \left( \hat{\beta^{\left( i \right)}}-\beta\right),\quad\quad$$

$$\text{MSE}\left( \hat{\beta} \right)=\frac{1}{R}\sum_{i=1}^{R} \left( \hat{\beta^{\left( i \right)}}-\beta\right)^{2}.$$

**Location:** Section **7 (Simulation Study)**, notation paragraph and formulas updated.

**14. “What are a and b in Table 4?”**

**Response:** We standardized parameter notation throughout to $\alpha$ and $\beta$. Table 4 caption and column headers were updated to read “$\alpha$” and “$\beta$”; we added a footnote to Table 4 specifying the true parameter values used for each block and that **R = 1000** Monte Carlo replicates were used (this was the replicate count used in our study).
**Location:** Table 4 (caption and footnote) and the paragraph describing simulation settings (Section **7**).

**15. “What is the purpose of TTT plot? How is it drawn?”**

**Response:** We added a brief description of the Total Time on Test (TTT) transform in Section **6** (Data Exploratory Diagnostics) explaining its purpose and interpretation (convex → increasing hazard, concave → decreasing hazard, concave→convex → bathtub). We also included the formula used and the R code used to compute and plot the TTT curve in Supporting File . **Fig 4** (TTT plot) is retained and its caption expanded to describe interpretation for our data.
**Location:** Section **6**, paragraph on exploratory diagnostics; Supporting file contains R code.

**16. “Present the elements of the Hessian matrix in Section 4. Argue whether it is negative definite?”**

**Response:** We added:

- The analytic second-derivative forms (symbolic expressions) where compactly expressible (Section **4.1**, second derivatives). Where expressions become lengthy we provide the functional forms and refer to the numeric evaluation.
- A numerical evaluation of the Hessian (observed information matrix) at the MLE for the COVID-19 data is now included (numeric matrix printed in the text) and the eigenvalues of the observed Fisher information are displayed. The eigenvalues demonstrate the expected definiteness (the Hessian of the log-likelihood is negative definite at the MLE; equivalently, the Hessian of the negative log-likelihood is positive definite), supporting that the reported MLE is a local maximum.
- The R code used to compute the numeric Hessian using numDeriv::hessian() and the optim() MLE routine is included in Supporting File .

**Location:** Section **4.1** (second derivative expressions and numerical Hessian evaluation) and Appendix A (numeric Hessian + eigenvalues and a short interpretative remark).

**17. “English language usage shall be verified.”**

**Response:** We proofread and copy-edited the manuscript to improve grammar and clarity (every section revised for readability). We fixed typographical errors, ambiguous phrasing, and standardized mathematical notation. (If the reviewers or editor would like, we can supply a tracked-changes version showing all language edits; we are uploading one as requested.)
**Location:** Global—tracked-changes manuscript uploaded.

**18. “References shall be cited in uniform format.”**

**Response:** We standardized the references to a uniform PLOS-compatible style (numbered in order of appearance) and fixed duplicate citations. The bibliography has been reformatted and checked for completeness.
**Location:** References section (entire reference list updated).

**Responses to Reviewer #2**

Reviewer 2 found the manuscript original and methodologically sound; requested minor clarifications for clarity, reproducibility, and discussion of limitations.

We thank Reviewer 2 for the positive evaluation. We have implemented minor clarifications and improved reproducibility.

**1. Clarity / Reproducibility**

**Response:** We added:

- A short paragraph in **Materials and Methods** describing the software used (R version 4.5.0 , and the scripts included in Supporting File.
- All simulation and estimation code as Supporting File (R scripts), with comments and instructions for reproducing tables and figures.

**Location:** Methods (Reproducibility subsection) and Supporting file.

*Note to authors:* please confirm the exact R version and package versions to put in the manuscript.

**2. Discussion of limitations**

**Response:** We expanded the Discussion (final paragraph) to discuss limitations including: (i) analysis performed on uncensored data (extension to censored data requires EM or partial likelihood methods), (ii) moderate sample size (n=106) in the COVID-19 example, and (iii) generalisability to other populations (differences in reporting across locations). We also suggested immediate extensions (incorporate covariates, right-censoring, and Bayesian estimation).
**Location:** Discussion section (new paragraph on limitations and future work).

**Final Remarks**

We believe that our revisions, guided by the reviewers' insightful comments, have substantially improved the manuscript's clarity, rigor, and reproducibility. We are grateful for the opportunity to revise our work and hope that it is now deemed suitable for publication in PLOS ONE.

Sincerely,
Abdulrahman O. Alshammari(CA), Hadi O. Alshammari, Hameed Ali
