# Supplementary material for: A flexible exponential type family for modeling non-monotonic hazard rates with application to mortality analysis: A COVID-19 case study
Source: PLoS One. 2026 Aug 6;21(8):e0331050. doi: 10.1371/journal.pone.0331050 (PMC13446746; doi:10.1371/journal.pone.0331050)
Supplement: Supplementary file 5 [file pone.0331050.s005.docx]

**Response to Reviewers**

**Manuscript ID:** PONE-D-25-43305R1

**Title:** *A flexible exponential-type family for modeling non-monotonic hazard rates with application to mortality analysis: A COVID-19 case study*

**Dear Editor,**

We sincerely thank you for your careful evaluation of our revised manuscript and for your encouraging remarks. We are grateful for the opportunity to submit a final revised version.

We are pleased that the manuscript has been found suitable for publication pending minor revisions. We have carefully addressed all the points raised and provide a detailed, point-by-point response below.

Editor’s Comments and Responses

**Comment 1:** *Perform a final careful proofreading to ensure clarity, grammatical correctness, and consistency throughout the manuscript.*

**Response:** We thank the Editor for this suggestion. The manuscript has undergone a thorough and careful proofreading to enhance clarity, grammatical accuracy, and overall readability.

Specifically, sentence structures have been refined to improve flow and precision, minor grammatical inconsistencies have been corrected throughout the manuscript. terminology and notation have been standardized to ensure consistency across all sections, and redundant and informal expressions have been removed or replaced with more formal academic phrasing.

These revisions have improved the overall presentation and readability of the manuscript.

**Comment 2:** *Confirm and clearly state the exact version(s) of R and any packages used in the analysis.*

**Response:** We appreciate this important suggestion regarding reproducibility. The manuscript has been revised to explicitly include details of the computational environment used in the analysis.

A dedicated statement has been added (Section 7) specifying the version of R used (*R version 4.3.2*), the optimization procedure (optim() function with the Nelder–Mead algorithm), Supporting tools for numerical differentiation (e.g., numDeriv) , and visualization tools ( ggplot2). This addition ensures full transparency and reproducibility of the results.

**Comment 3:** *Ensure that all figures, tables, and references strictly conform to journal formatting guidelines.*

**Response:** We thank the Editor for highlighting this important point. A comprehensive formatting review has been conducted across the entire manuscript.

The following improvements have been implemented:

- All figures and tables have been renumbered and formatted consistently according to journal guidelines.
- Figure captions have been revised for clarity and uniform structure.
- Table titles and alignments have been standardized.
- Equation numbering has been corrected and cross-referenced consistently throughout the text.
- References have been carefully checked for completeness, consistency, and proper formatting, including DOI presentation where applicable.

These revisions ensure full compliance with the journal’s formatting requirements. In addition to the above revisions, minor improvements were made to enhance clarity in selected sections (e.g., Abstract, Methodology, and Simulation Study), without altering the scientific content or conclusions of the manuscript.

We believe that the manuscript has been further strengthened through these final refinements and now fully meets the journal’s publication standards.

We sincerely thank the Editor for the constructive feedback and for the opportunity to improve our work. We look forward to your final decision.

**Sincerely,**

**Dr. Bilal Himmat(CA)**

On behalf of all authors
