# Supplementary material for: A flexible exponential type family for modeling non-monotonic hazard rates with application to mortality analysis: A COVID-19 case study
Source: PLoS One. 2026 Aug 6;21(8):e0331050. doi: 10.1371/journal.pone.0331050 (PMC13446746; doi:10.1371/journal.pone.0331050)
Supplement: Peer review history

# Peer review: Decision Letter 0

**DOI:** 10.1371/journal.pone.0331050.r001

**Authors:** Shaiful Anuar Abu Bakar

10 Oct 2025

PONE-D-25-43305

Innovative Modeling of Non-Monotonic Hazard Rates: A Flexible Exponential Type Family Approach with Application to COVID-19 Mortality Analysis

PLOS ONE

Dear Dr. Alshammari,

Thank you for submitting your manuscript to PLOS ONE. After careful consideration, we feel that it has merit but does not fully meet PLOS ONE’s publication criteria as it currently stands. Therefore, we invite you to submit a revised version of the manuscript that addresses the points raised during the review process.

Please submit your revised manuscript by Nov 24 2025 11:59PM. If you will need more time than this to complete your revisions, please reply to this message or contact the journal office at plosone@plos.org. When you're ready to submit your revision, log on to https://www.editorialmanager.com/pone/ and select the 'Submissions Needing Revision' folder to locate your manuscript file.

Please include the following items when submitting your revised manuscript:

- A rebuttal letter that responds to each point raised by the academic editor and reviewer(s). You should upload this letter as a separate file labeled 'Response to Reviewers'.
- A marked-up copy of your manuscript that highlights changes made to the original version. You should upload this as a separate file labeled 'Revised Manuscript with Track Changes'.
- An unmarked version of your revised paper without tracked changes. You should upload this as a separate file labeled 'Manuscript'.

-->If you would like to make changes to your financial disclosure, please include your updated statement in your cover letter. Guidelines for resubmitting your figure files are available below the reviewer comments at the end of this letter.

If applicable, we recommend that you deposit your laboratory protocols in protocols.io to enhance the reproducibility of your results. Protocols.io assigns your protocol its own identifier (DOI) so that it can be cited independently in the future. For instructions see: https://journals.plos.org/plosone/s/submission-guidelines#loc-laboratory-protocols. Additionally, PLOS ONE offers an option for publishing peer-reviewed Lab Protocol articles, which describe protocols hosted on protocols.io. Read more information on sharing protocols at https://plos.org/protocols?utm_medium=editorial-email&utm_source=authorletters&utm_campaign=protocols.

We look forward to receiving your revised manuscript.

Kind regards,

Shaiful Anuar Abu Bakar, Ph.D., M.Sc.

Academic Editor

PLOS ONE

Journal Requirements:

When submitting your revision, we need you to address these additional requirements.

1. Please ensure that your manuscript meets PLOS ONE's style requirements, including those for file naming. The PLOS ONE style templates can be found at

https://journals.plos.org/plosone/s/file?id=wjVg/PLOSOne_formatting_sample_main_body.pdf and https://journals.plos.org/plosone/s/file?id=ba62/PLOSOne_formatting_sample_title_authors_affiliations.pdf

2. Please note that PLOS One has specific guidelines on code sharing for submissions in which author-generated code underpins the findings in the manuscript. In these cases, we expect all author-generated code to be made available without restrictions upon publication of the work. Please review our guidelines at https://journals.plos.org/plosone/s/materials-and-software-sharing#loc-sharing-code and ensure that your code is shared in a way that follows best practice and facilitates reproducibility and reuse.

3. Thank you for stating the following in the Acknowledgments Section of your manuscript:

[This work was funded by the Deanship of Graduate Studies and Scientific Research at Jouf University under grant No. (DGSSR-2025-NF-02-053)]

We note that you have provided funding information that is not currently declared in your Funding Statement. However, funding information should not appear in the Acknowledgments section or other areas of your manuscript. We will only publish funding information present in the Funding Statement section of the online submission form.

Please remove any funding-related text from the manuscript and let us know how you would like to update your Funding Statement. Currently, your Funding Statement reads as follows:

[The author(s) received no specific funding for this work.]

Please include your amended statements within your cover letter; we will change the online submission form on your behalf.

4. In the online submission form, you indicated that [Data cannot be shared publicly because they are owned by Mexico’s Ministry of Health and contain sensitive patient information. Data are available from the Ministry of Health (https://www.gob.mx/salud) for researchers who meet the criteria for access to confidential data.].

All PLOS journals now require all data underlying the findings described in their manuscript to be freely available to other researchers, either 1. In a public repository, 2. Within the manuscript itself, or 3. Uploaded as supplementary information.

This policy applies to all data except where public deposition would breach compliance with the protocol approved by your research ethics board. If your data cannot be made publicly available for ethical or legal reasons (e.g., public availability would compromise patient privacy), please explain your reasons on resubmission and your exemption request will be escalated for approval.

5. Please ensure that you refer to Figure Fig-1 in your text as, if accepted, production will need this reference to link the reader to the figure.

6. Please upload a copy of Figure 2, to which you refer in your text on pages 5 and 7. If the figure is no longer to be included as part of the submission please remove all reference to it within the text.

If the reviewer comments include a recommendation to cite specific previously published works, please review and evaluate these publications to determine whether they are relevant and should be cited. There is no requirement to cite these works unless the editor has indicated otherwise.

Please review your reference list to ensure that it is complete and correct. If you have cited papers that have been retracted, please include the rationale for doing so in the manuscript text, or remove these references and replace them with relevant current references. Any changes to the reference list should be mentioned in the rebuttal letter that accompanies your revised manuscript. If you need to cite a retracted article, indicate the article’s retracted status in the References list and also include a citation and full reference for the retraction notice.

Additional Editor Comments:

Before resubmitting, please review the two reviewers' remarks and make necessary refinement based on the comments.

[Note: HTML markup is below. Please do not edit.]

Reviewers' comments:

Reviewer's Responses to Questions

**Comments to the Author**

1. Is the manuscript technically sound, and do the data support the conclusions?

The manuscript must describe a technically sound piece of scientific research with data that supports the conclusions. Experiments must have been conducted rigorously, with appropriate controls, replication, and sample sizes. The conclusions must be drawn appropriately based on the data presented.

Reviewer #1: Yes

Reviewer #2: Yes

**********

2. Has the statistical analysis been performed appropriately and rigorously?

Reviewer #1: Yes

Reviewer #2: Yes

**********

3. Have the authors made all data underlying the findings in their manuscript fully available?

The PLOS Data policy requires authors to make all data underlying the findings described in their manuscript fully available without restriction, with rare exception (please refer to the Data Availability Statement in the manuscript PDF file). The data should be provided as part of the manuscript or its supporting information, or deposited to a public repository. For example, in addition to summary statistics, the data points behind means, medians and variance measures should be available. If there are restrictions on publicly sharing data—e.g. participant privacy or use of data from a third party—those must be specified.

Reviewer #1: Yes

Reviewer #2: Yes

**********

4. Is the manuscript presented in an intelligible fashion and written in standard English?

PLOS ONE does not copyedit accepted manuscripts, so the language in submitted articles must be clear, correct, and unambiguous. Any typographical or grammatical errors should be corrected at revision, so please note any specific errors here.

Reviewer #1: No

Reviewer #2: Yes

**********

5. Review Comments to the Author

Please use the space provided to explain your answers to the questions above. You may also include additional comments for the author, including concerns about dual publication, research ethics, or publication ethics. (Please upload your review as an attachment if it exceeds 20,000 characters)

Reviewer #1: Reviewer’s Comments on the manuscript entitled

Innovative Modeling of Non-Monotonic Hazard Rates: A Flexible Exponential Type Family Approach with Application to COVID-19 Mortality Analysis

Observations:

A new family of distributions for survival analysis experiments is introduced in this paper to accommodate non-monotonic pattern of hazard rates. It is a practically valid situation. Statistical properties of the distribution is studied. Application of the distribution to a real data on COVID-19 mortality rates is attempted. Appropriateness of the MLEs is discussed through simulation studies.

Comments:

However, clarifications and editing are required on the following:

1. Explain the rationale behind the expression for the CDF presented in equation (1).

2. Though it is obvious in survival models, mention the support of the random variable.

3. Why there is no shape / location parameter?

4. Equation number (3) is repeated.

5. Equation numbers shall be typed in appropriate places. For instance, equation – (6).

6. Figure numbers, from Fig.1, are not in order.

7. The parameters a and b are not included in MRL expression in equation (8).

8. Expressions for f(x) and R(x) are repeated.

9. In Section 3.4, it should be “ the PDF of the smallest order statistic … “ and “ the PDF of the largest order statistic … “.

10. Whether the liklelihood function is bounded with respect to both the parameters? Present graphs of the liklelihood function.

11. Do the mortality rate presented in Section 6 correspond to randomly selected days’ or randomly selected locations of Mexico? Mention the authenticity of the data. Also, mention whether the mortality rate is per 1,000 or 10,000?

12. What is the basis on which the distributions Wb, TIW, IW and APW are considered as competing distributions against ETW distribution?

13. What are K, βi and β in the expressions of MSE and Bias?

14. What are a and b in Table 4?

15. What is the purpose of TTT plot? How is it drawn?

16. Present the elements of the Hessian matrix in Section 4. Argue whether it is negative definite?

17. English language usage shall be verified.

18. References shall be cited in uniform format.

Reviewer #2: The manuscript meets the criteria for originality, methodological rigor, and significance. It contributes valuable new tools for survival analysis with broad applications. Minor revisions for clarity, reproducibility, and discussion of limitations are recommended to fully align with PLOS ONE standards.

**********

6. PLOS authors have the option to publish the peer review history of their article (what does this mean?). If published, this will include your full peer review and any attached files.

If you choose “no”, your identity will remain anonymous but your review may still be made public.

**Do you want your identity to be public for this peer review?**  For information about this choice, including consent withdrawal, please see our Privacy Policy.

Reviewer #1: No

Reviewer #2: **Yes:** Kenenisa Abdisa Kuse

**********

[NOTE: If reviewer comments were submitted as an attachment file, they will be attached to this email and accessible via the submission site. Please log into your account, locate the manuscript record, and check for the action link "View Attachments". If this link does not appear, there are no attachment files.]

While revising your submission, please upload your figure files to the Preflight Analysis and Conversion Engine (PACE) digital diagnostic tool, https://pacev2.apexcovantage.com/. PACE helps ensure that figures meet PLOS requirements. To use PACE, you must first register as a user. Registration is free. Then, login and navigate to the UPLOAD tab, where you will find detailed instructions on how to use the tool. If you encounter any issues or have any questions when using PACE, please email PLOS at figures@plos.org. Please note that Supporting Information files do not need this step.

# Author comment: Author response to Decision Letter 1

**DOI:** 10.1371/journal.pone.0331050.r002

20 Dec 2025

Response to Reviewers: PONE-D-25-43305

Title: Innovative Modeling of Non-Monotonic Hazard Rates: A Flexible Exponential Type Family Approach with Application to COVID-19 Mortality Analysis

Authors: Abdulrahman O. Alshammari, Hadi O. Alshammari, Hameed Ali

We thank the Academic Editor and the Reviewers for their thorough and constructive comments. Below we respond point-by-point to every comment and indicate the changes made in the revised manuscript (tracked changes version). Where we added new equations, figures, tables, code, or text, we cite the section and paragraph number in the revised manuscript.

Responses to Reviewer #1

Reviewer 1 requested clarifications on CDF rationale, support, notation (a/b), numbering and formatting issues, MRL, duplicate expressions, order statistics language, likelihood behaviour, data provenance & units, choices of competing models, notation in simulation formulas, Table 4 parameter labels, purpose of TTT plot, Hessian elements and definiteness, English editing and uniform reference format.

We thank Reviewer 1 for constructive, detailed comments. We have addressed each below and revised the manuscript accordingly.

1. “Explain the rationale behind the expression for the CDF presented in equation (1).”

Response: We added a short subsection immediately after Equation (1) that explains the generator used to construct the Flexible Exponential-Type Family (FETF) and how the ETW (Exponential-Type Weibull) CDF arises as the special case of the general form. The new text explains the outer exponential transform, the baseline Weibull CDF used, and the intended properties (boundedness [0,1], exponential tail behavior, and flexibility to generate non-monotonic hazard shapes).

Location (revised manuscript): Section 2 (after eq. (1)), paragraph added titled “Rationale / construction of the CDF”.

“The FETF is constructed by applying an exponential-type transform to a baseline CDF G(x;\theta). Specifically, the ETW arises by taking (G) as the Weibull CDF and applying the transform F(x)=1-\exp\funcapply{-\lambda H(G(x))}\ with\ (H(\cdot))\ =\ \ldots\ (explicit form used). This ensures F(0)=0,\ F(\infty)=1, and introduces the required flexibility while maintaining closed-form expressions for many properties.”

2. “Though it is obvious in survival models, mention the support of the random variable.”

Response: We added an explicit statement of support where the random variable is first introduced.

Location: Section 2, first paragraph.

Text added: “Let X be a nonnegative continuous random variable with support x\in\left(0,\infty\right).”

3. “Why there is no shape / location parameter?”

Response: We added a short justification for the two-parameter specification (parsimonious modeling rationale) and commented that location or additional shape parameters could be included in future extensions but were intentionally omitted to keep estimation stable for moderate sample sizes.

Location: End of Section 2 (model definition paragraph).

Text added: “We chose a parsimonious two-parameter formulation (parameters \alpha,\beta) to balance flexibility and interpretability; introducing additional location or shape parameters increases estimation complexity and can reduce stability for moderate sample sizes. Extensions that include such parameters are straightforward and are left for future work.”

4. “Equation number (3) is repeated.”

Response: We carefully renumbered all equations sequentially and checked all in-text cross-references. There are no duplicate equation numbers in the revised manuscript. (All cross-references were updated as needed.)

Location: Global (equation numbering corrected across Sections 2–5).

5. “Equation numbers shall be typed in appropriate places. For instance, equation – (6).”

Response: We reviewed and fixed all placement and formatting of equation numbers (all parentheses and labels follow PLOS style). All in-text references to equation numbers have been updated to reflect the new numbering.

Location: Global (Sections 2–5).

6. “Figure numbers, from Fig.1, are not in order.”

Response: We reordered figures so that figure numbers follow the order of their first appearance in the text and updated all figure references to use the format “Fig 1”, “Fig 2”, etc. We also fixed caption formatting and ensured each figure file is uploaded separately in high resolution.

Location: Figures and their first mention in Introduction/Results.

7. “The parameters a and b are not included in MRL expression in equation (8).”

Response: We reviewed the derivation for the mean residual life (MRL) and corrected the expression so that the parameters (now consistently denoted \alpha and \beta) are explicitly present in the MRL formulas. The derivation steps were expanded and the final expression displays parameter dependence explicitly.

Location: Section 3.2 (Mean Residual Life); Equation updated (previously eq.(8), renumbered in revised manuscript).

8. “Expressions for f(x) and R(x) are repeated.”

Response: Duplicate expressions were removed; a single, clearly labelled PDF f(x) and survival function S(x) (or R(x) where used) are retained and cross-referenced.

Location: Section 2 (model definition) and Section 3.1 (survival & hazard).

9. “In Section 3.4, it should be 'the PDF of the smallest order statistic …' and 'the PDF of the largest order statistic …'.”

Response: Wording corrected and formulas rechecked for order statistics. We replaced ambiguous phrasing with precise terms “smallest (first) order statistic X_{\left(1\right)})” and “largest (nth) order statistic X_{\left(n\right)}”. Minor typographical fixes applied.

Location: Section 3.4 (Order statistics).

10. “Whether the likelihood function is bounded with respect to both the parameters? Present graphs of the likelihood function.”

Response: We added a new subsection 4.2 Profile and Surface Plots of the Log-Likelihood:

We compute the log-likelihood surface over a grid of ((\alpha,\beta) values and present (i) contour plot, (ii) perspective plot, and (iii) one-dimensional profile log-likelihoods for each parameter holding the other at its MLE. These figures demonstrate the log-likelihood is bounded and displays a clear maximum for the COVID-19 dataset and for representative simulated datasets.

We included the R code used to generate these figures as Supporting File so reviewers can reproduce the plots.

Location: Section 4.2 (new). Figures added: Fig 6 (likelihood contour) and Fig 7 (profile likelihood plots). R code in Supporting file.

11. “Do the mortality rate presented in Section 6 correspond to randomly selected days’ or randomly selected locations of Mexico? Mention the authenticity of the data. Also, mention whether the mortality rate is per 1,000 or 10,000?”

Response: We clarified the origin and nature of the dataset in Section 6 (Real Data Application / Data Set). The dataset contains 106 anonymized observations labeled in the manuscript; each entry corresponds to the reported mortality rate measure as provided in the original Ministry of Health file.

Location: Section 6 (Data Set: Mortality Rate), paragraph expanded. The 106 numeric values are also listed in the revised manuscript (unchanged), and an aggregated summary table (mean, median, SD, min, max) has been added.

The manuscript now contains an explicit description of the variable unit and provenance. If you prefer a controlled-access wording (if there are restrictions), we kept the Data Availability statement consistent with the editor’s instruction to escalate any exemption requests.

12. “What is the basis on which the distributions Wb, TIW, IW and APW are considered as competing distributions against ETW distribution?”

Response: We added justification and citations for each competing model in Section 6 (Real Data Application):

Weibull (Wb): standard two-parameter baseline model.

Inverse Weibull (IW): flexible for heavy-tailed lifetimes; widely used in reliability.

Transmuted Inverse Weibull (TIW): introduces extra shape flexibility and has been applied in similar mortality and reliability contexts.

Alpha-Power Weibull (APW): commonly used flexible three-parameter family in biomedical applications (cited recent literature).

We also added citations to recent COVID-19 mortality modeling studies that used these distributions (references added in the revised manuscript).

Location: Section 6, paragraph introducing competing models (we added brief rationale and references).

13. “What are K, βi and β in the expressions of MSE and Bias?”

Response: We clarified notation in the simulation section. We replaced ambiguous symbols with standard notation and explicitly defined them:

R (previously K in some drafts) denotes the number of Monte Carlo replicates (we used R=1000 except where otherwise specified).

\widehat{\beta^{\left(i\right)}} denotes the estimate in the (i)-th Monte Carlo replication; \beta denotes the true parameter value.

We included the standard formulae for Monte Carlo Bias and MSE:

\mathrm{Bias}\left(\hat{\beta}\right)=\frac{1}{R}\sum_{i=1}^{R}\left(\widehat{\beta^{\left(i\right)}}-\beta\right),\emsp\emsp\bigm\mathrm{MSE}\left(\hat{\beta}\right)=\frac{1}{R}\sum_{i=1}^{R}\left(\widehat{\beta^{\left(i\right)}}-\beta\right)^2.

Location: Section 7 (Simulation Study), notation paragraph and formulas updated.

14. “What are a and b in Table 4?”

Response: We standardized parameter notation throughout to \alpha and \beta. Table 4 caption and column headers were updated to read “\alpha” and “\beta”; we added a footnote to Table 4 specifying the true parameter values used for each block and that R = 1000 Monte Carlo replicates were used (this was the replicate count used in our study).

Location: Table 4 (caption and footnote) and the paragraph describing simulation settings (Section 7).

15. “What is the purpose of TTT plot? How is it drawn?”

Response: We added a brief description of the Total Time on Test (TTT) transform in Section 6 (Data Exploratory Diagnostics) explaining its purpose and interpretation (convex → increasing hazard, concave → decreasing hazard, concave→convex → bathtub). We also included the formula used and the R code used to compute and plot the TTT curve in Supporting File . Fig 4 (TTT plot) is retained and its caption expanded to describe interpretation for our data.

Location: Section 6, paragraph on exploratory diagnostics; Supporting file contains R code.

16. “Present the elements of the Hessian matrix in Section 4. Argue whether it is negative definite?”

Response: We added:

The analytic second-derivative forms (symbolic expressions) where compactly expressible (Section 4.1, second derivatives). Where expressions become lengthy we provide the functional forms and refer to the numeric evaluation.

A numerical evaluation of the Hessian (observed information matrix) at the MLE for the COVID-19 data is now included (numeric matrix printed in the text) and the eigenvalues of the observed Fisher information are displayed. The eigenvalues demonstrate the expected definiteness (the Hessian of the log-likelihood is negative definite at the MLE; equivalently, the Hessian of the negative log-likelihood is positive definite), supporting that the reported MLE is a local maximum.

The R code used to compute the numeric Hessian using numDeriv::hessian() and the optim() MLE routine is included in Supporting File .

Location: Section 4.1 (second derivative expressions and numerical Hessian evaluation) and Appendix A (numeric Hessian + eigenvalues and a short interpretative remark).

17. “English language usage shall be verified.”

Response: We proofread and copy-edited the manuscript to improve grammar and clarity (every section revised for readability). We fixed typographical errors, ambiguous phrasing, and standardized mathematical notation. (If the reviewers or editor would like, we can supply a tracked-changes version showing all language edits; we are uploading one as requested.)

Location: Global—tracked-changes manuscript uploaded.

18. “References shall be cited in uniform format.”

Response: We standardized the references to a uniform PLOS-compatible style (numbered in order of appearance) and fixed duplicate citations. The bibliography has been reformatted and checked for completeness.

Location: References section (entire reference list updated).

Responses to Reviewer #2

Reviewer 2 found the manuscript original and methodologically sound; requested minor clarifications for clarity, reproducibility, and discussion of limitations.

We thank Reviewer 2 for the positive evaluation. We have implemented minor clarifications and improved reproducibility.

1. Clarity / Reproducibility

Response: We added:

A short paragraph in Materials and Methods describing the software used (R version 4.5.0 , and the scripts included in Supporting File.

All simulation and estimation code as Supporting File (R scripts), with comments and instructions for reproducing tables and figures.

Location: Methods (Reproducibility subsection) and Supporting file.

Note to authors: please confirm the exact R version and package versions to put in the manuscript.

2. Discussion of limitations

Response: We expanded the Discussion (final paragraph) to discuss limitations including: (i) analysis performed on uncensored data (extension to censored data requires EM or partial likelihood methods), (ii) moderate sample size (n=106) in the COVID-19 example, and (iii) generalisability to other populations (differences in reporting across locations). We also suggested immediate extensions (incorporate covariates, right-censoring, and Bayesian estimation).

Location: Discussion section (new paragraph on limitations and future work).

Final Remarks

We believe that our revisions, guided by the reviewers' insightful comments, have substantially improved the manuscript's clarity, rigor, and reproducibility. We are grateful for the opportunity to revise our work and hope that it is now deemed suitable for publication in PLOS ONE.

Sincerely,

Abdulrahman O. Alshammari(CA), Hadi O. Alshammari, Hameed Ali

Attachment Submitted filename: Response to the reviewers comments.docx

# Peer review: Decision Letter 1

**DOI:** 10.1371/journal.pone.0331050.r003

**Authors:** Umair Khalil

17 Mar 2026

PONE-D-25-43305R1

A Flexible Exponential Type Family for Modeling Non-Monotonic Hazard Rates with Application Mortality Analysis: A COVID-19 Case Study

PLOS One

Dear Dr. Himmat,

Thank you for submitting your manuscript to PLOS ONE. After careful consideration, we feel that it has merit but does not fully meet PLOS ONE’s publication criteria as it currently stands. Therefore, we invite you to submit a revised version of the manuscript that addresses the points raised during the review process.

==============================

After careful evaluation of the revised manuscript and your detailed responses to the reviewers, I am pleased to inform you that your manuscript is **accepted pending minor revisions** .

Both reviewers’ comments have been addressed thoroughly and thoughtfully. In particular, the manuscript has been significantly improved in terms of:

- Clarity of model construction and theoretical justification,
- Consistency of notation and correction of technical issues,
- Inclusion of additional analyses (e.g., likelihood surfaces, Hessian evaluation),
- Improved reproducibility through provision of code and methodological details,
- Expanded discussion, including limitations and practical implications.

Before final acceptance, please address the following **minor points** :

1. **Language and Formatting:**
  Perform a final careful proofreading to ensure clarity, grammatical correctness, and consistency throughout the manuscript.
2. **Software Details:**
  Confirm and clearly state the exact version(s) of R and any packages used in the analysis.
3. **Final Formatting Check:**
  Ensure that all figures, tables, and references strictly conform to journal formatting guidelines.

Once these minor revisions are completed, your manuscript will be ready for final acceptance and publication.

We appreciate your careful and thorough revisions, and we thank you for choosing **PLOS ONE**  for the dissemination of your work.

==============================

Please submit your revised manuscript by May 01 2026 11:59PM. If you will need more time than this to complete your revisions, please reply to this message or contact the journal office at plosone@plos.org. When you're ready to submit your revision, log on to https://www.editorialmanager.com/pone/ and select the 'Submissions Needing Revision' folder to locate your manuscript file.

Please include the following items when submitting your revised manuscript:

- A letter that responds to each point raised by the academic editor and reviewer(s). You should upload this letter as a separate file labeled 'Response to Reviewers'.
- A marked-up copy of your manuscript that highlights changes made to the original version. You should upload this as a separate file labeled 'Revised Manuscript with Track Changes'.
- An unmarked version of your revised paper without tracked changes. You should upload this as a separate file labeled 'Manuscript'.

-->If you would like to make changes to your financial disclosure, please include your updated statement in your cover letter. Guidelines for resubmitting your figure files are available below the reviewer comments at the end of this letter.

If applicable, we recommend that you deposit your laboratory protocols in protocols.io to enhance the reproducibility of your results. Protocols.io assigns your protocol its own identifier (DOI) so that it can be cited independently in the future. For instructions see: https://journals.plos.org/plosone/s/submission-guidelines#loc-laboratory-protocols. Additionally, PLOS ONE offers an option for publishing peer-reviewed Lab Protocol articles, which describe protocols hosted on protocols.io. Read more information on sharing protocols at https://plos.org/protocols?utm_medium=editorial-email&utm_source=authorletters&utm_campaign=protocols.

We look forward to receiving your revised manuscript.

Kind regards,

Umair Khalil, PhD

Academic Editor

PLOS One

Journal Requirements:

If the reviewer comments include a recommendation to cite specific previously published works, please review and evaluate these publications to determine whether they are relevant and should be cited. There is no requirement to cite these works unless the editor has indicated otherwise.

Please review your reference list to ensure that it is complete and correct. If you have cited papers that have been retracted, please include the rationale for doing so in the manuscript text, or remove these references and replace them with relevant current references. Any changes to the reference list should be mentioned in the rebuttal letter that accompanies your revised manuscript. If you need to cite a retracted article, indicate the article’s retracted status in the References list and also include a citation and full reference for the retraction notice.

Additional Editor Comments:

Dear Authors,

Thank you for submitting the revised version of your manuscript entitled:

“Innovative Modeling of Non-Monotonic Hazard Rates: A Flexible Exponential Type Family Approach with Application to COVID-19 Mortality Analysis” (PONE-D-25-43305)

to PLOS ONE.

After careful evaluation of the revised manuscript and your detailed responses to the reviewers, I am pleased to inform you that your manuscript is accepted pending minor revisions.

Both reviewers’ comments have been addressed thoroughly and thoughtfully. In particular, the manuscript has been significantly improved in terms of:

Clarity of model construction and theoretical justification,

Consistency of notation and correction of technical issues,

Inclusion of additional analyses (e.g., likelihood surfaces, Hessian evaluation),

Improved reproducibility through provision of code and methodological details,

Expanded discussion, including limitations and practical implications.

Before final acceptance, please address the following minor points:

Language and Formatting:

Perform a final careful proofreading to ensure clarity, grammatical correctness, and consistency throughout the manuscript.

Software Details:

Confirm and clearly state the exact version(s) of R and any packages used in the analysis.

Final Formatting Check:

Ensure that all figures, tables, and references strictly conform to journal formatting guidelines.

Once these minor revisions are completed, your manuscript will be ready for final acceptance and publication.

We appreciate your careful and thorough revisions, and we thank you for choosing PLOS ONE for the dissemination of your work.

[Note: HTML markup is below. Please do not edit.]

Reviewers' comments:

Reviewer's Responses to Questions

**Comments to the Author**

1. If the authors have adequately addressed your comments raised in a previous round of review and you feel that this manuscript is now acceptable for publication, you may indicate that here to bypass the “Comments to the Author” section, enter your conflict of interest statement in the “Confidential to Editor” section, and submit your "Accept" recommendation.

Reviewer #1: All comments have been addressed

Reviewer #3: All comments have been addressed

Reviewer #4: All comments have been addressed

Reviewer #5: All comments have been addressed

**********

2. Is the manuscript technically sound, and do the data support the conclusions?

The manuscript must describe a technically sound piece of scientific research with data that supports the conclusions. Experiments must have been conducted rigorously, with appropriate controls, replication, and sample sizes. The conclusions must be drawn appropriately based on the data presented.

Reviewer #1: Yes

Reviewer #3: Yes

Reviewer #4: Yes

Reviewer #5: Yes

**********

3. Has the statistical analysis been performed appropriately and rigorously?

Reviewer #1: Yes

Reviewer #3: Yes

Reviewer #4: Yes

Reviewer #5: Yes

**********

4. Have the authors made all data underlying the findings in their manuscript fully available?

The PLOS Data policy requires authors to make all data underlying the findings described in their manuscript fully available without restriction, with rare exception (please refer to the Data Availability Statement in the manuscript PDF file). The data should be provided as part of the manuscript or its supporting information, or deposited to a public repository. For example, in addition to summary statistics, the data points behind means, medians and variance measures should be available. If there are restrictions on publicly sharing data—e.g. participant privacy or use of data from a third party—those must be specified.

Reviewer #1: No

Reviewer #3: Yes

Reviewer #4: Yes

Reviewer #5: Yes

**********

5. Is the manuscript presented in an intelligible fashion and written in standard English?

PLOS ONE does not copyedit accepted manuscripts, so the language in submitted articles must be clear, correct, and unambiguous. Any typographical or grammatical errors should be corrected at revision, so please note any specific errors here.

Reviewer #1: Yes

Reviewer #3: No

Reviewer #4: Yes

Reviewer #5: Yes

**********

6. Review Comments to the Author

Please use the space provided to explain your answers to the questions above. You may also include additional comments for the author, including concerns about dual publication, research ethics, or publication ethics. (Please upload your review as an attachment if it exceeds 20,000 characters)

Reviewer #1: (No Response)

Reviewer #3: There are certain typo, missing commas, illogical creation of section and sub section. So address these points.

Reviewer #4: The authors have addressed all my comments and I therefore recommend for the acceptance of the manuscript.

Reviewer #5: (No Response)

**********

7. PLOS authors have the option to publish the peer review history of their article (what does this mean?). If published, this will include your full peer review and any attached files.

If you choose “no”, your identity will remain anonymous but your review may still be made public.

**Do you want your identity to be public for this peer review?**  For information about this choice, including consent withdrawal, please see our Privacy Policy.

Reviewer #1: No

Reviewer #3: No

Reviewer #4: No

Reviewer #5: No

**********

[NOTE: If reviewer comments were submitted as an attachment file, they will be attached to this email and accessible via the submission site. Please log into your account, locate the manuscript record, and check for the action link "View Attachments". If this link does not appear, there are no attachment files.]

To ensure your figures meet our technical requirements, please review our figure guidelines: https://journals.plos.org/plosone/s/figures

You may also use PLOS’s free figure tool, NAAS, to help you prepare publication quality figures: https://journals.plos.org/plosone/s/figures#loc-tools-for-figure-preparation.

NAAS will assess whether your figures meet our technical requirements by comparing each figure against our figure specifications.

# Author comment: Author response to Decision Letter 2

**DOI:** 10.1371/journal.pone.0331050.r004

22 Apr 2026

Response to Reviewers

Manuscript ID: PONE-D-25-43305R1

Title: A flexible exponential-type family for modeling non-monotonic hazard rates with application to mortality analysis: A COVID-19 case study

Dear Editor,

We sincerely thank you for your careful evaluation of our revised manuscript and for your encouraging remarks. We are grateful for the opportunity to submit a final revised version.

We are pleased that the manuscript has been found suitable for publication pending minor revisions. We have carefully addressed all the points raised and provide a detailed, point-by-point response below.

Editor’s Comments and Responses

Comment 1: Perform a final careful proofreading to ensure clarity, grammatical correctness, and consistency throughout the manuscript.

Response: We thank the Editor for this suggestion. The manuscript has undergone a thorough and careful proofreading to enhance clarity, grammatical accuracy, and overall readability.

Specifically, sentence structures have been refined to improve flow and precision, minor grammatical inconsistencies have been corrected throughout the manuscript. terminology and notation have been standardized to ensure consistency across all sections, and redundant and informal expressions have been removed or replaced with more formal academic phrasing.

These revisions have improved the overall presentation and readability of the manuscript.

Comment 2: Confirm and clearly state the exact version(s) of R and any packages used in the analysis.

Response: We appreciate this important suggestion regarding reproducibility. The manuscript has been revised to explicitly include details of the computational environment used in the analysis.

A dedicated statement has been added (Section 7) specifying the version of R used (R version 4.3.2), the optimization procedure (optim() function with the Nelder–Mead algorithm), Supporting tools for numerical differentiation (e.g., numDeriv) , and visualization tools ( ggplot2). This addition ensures full transparency and reproducibility of the results.

Comment 3: Ensure that all figures, tables, and references strictly conform to journal formatting guidelines.

Response: We thank the Editor for highlighting this important point. A comprehensive formatting review has been conducted across the entire manuscript.

The following improvements have been implemented:

• All figures and tables have been renumbered and formatted consistently according to journal guidelines.

• Figure captions have been revised for clarity and uniform structure.

• Table titles and alignments have been standardized.

• Equation numbering has been corrected and cross-referenced consistently throughout the text.

• References have been carefully checked for completeness, consistency, and proper formatting, including DOI presentation where applicable.

These revisions ensure full compliance with the journal’s formatting requirements. In addition to the above revisions, minor improvements were made to enhance clarity in selected sections (e.g., Abstract, Methodology, and Simulation Study), without altering the scientific content or conclusions of the manuscript.

We believe that the manuscript has been further strengthened through these final refinements and now fully meets the journal’s publication standards.

We sincerely thank the Editor for the constructive feedback and for the opportunity to improve our work. We look forward to your final decision.

Sincerely,

Dr. Bilal Himmat(CA)

On behalf of all authors

Attachment Submitted filename: Response to Reviewers.docx

# Peer review: Decision Letter 2

**DOI:** 10.1371/journal.pone.0331050.r005

**Authors:** Alessandro Barbiero

10 Jun 2026

PONE-D-25-43305R2

A flexible exponential type family for modeling non-monotonic hazard rates with application to mortality analysis: A COVID-19 case study

PLOS One

Dear Dr. Himmat,

Thank you for submitting your manuscript to PLOS ONE. After careful consideration, we feel that it has merit but does not fully meet PLOS ONE’s publication criteria as it currently stands. Therefore, we invite you to submit a revised version of the manuscript that addresses the points raised during the review process.

Please review Figure 2 and 3, since some (portions of the) curves fall outside the panel

Please submit your revised manuscript by Jul 25 2026 11:59PM. If you will need more time than this to complete your revisions, please reply to this message or contact the journal office at plosone@plos.org. When you're ready to submit your revision, log on to https://www.editorialmanager.com/pone/ and select the 'Submissions Needing Revision' folder to locate your manuscript file.

Please include the following items when submitting your revised manuscript:

- A letter that responds to each point raised by the academic editor and reviewer(s). You should upload this letter as a separate file labeled 'Response to Reviewers'.
- A marked-up copy of your manuscript that highlights changes made to the original version. You should upload this as a separate file labeled 'Revised Manuscript with Track Changes'.
- An unmarked version of your revised paper without tracked changes. You should upload this as a separate file labeled 'Manuscript'.

If you would like to make changes to your financial disclosure, please include your updated statement in your cover letter. Guidelines for resubmitting your figure files are available below the reviewer comments at the end of this letter.

If applicable, we recommend that you deposit your laboratory protocols in protocols.io to enhance the reproducibility of your results. Protocols.io assigns your protocol its own identifier (DOI) so that it can be cited independently in the future. For instructions see: https://journals.plos.org/plosone/s/submission-guidelines#loc-laboratory-protocols. Additionally, PLOS ONE offers an option for publishing peer-reviewed Lab Protocol articles, which describe protocols hosted on protocols.io. Read more information on sharing protocols at https://plos.org/protocols?utm_medium=editorial-email&utm_source=authorletters&utm_campaign=protocols.

As the corresponding author, your ORCID iD is verified in the submission system and will appear in the published article. PLOS supports the use of ORCID, and we encourage all coauthors to register for an ORCID iD and use it as well. Please encourage your coauthors to verify their ORCID iD within the submission system before final acceptance, as unverified ORCID iDs will not appear in the published article. *Only*  the individual author can complete the verification step; PLOS staff *cannot*  verify ORCID iDs on behalf of authors.

We look forward to receiving your revised manuscript.

Kind regards,

Alessandro Barbiero, Ph.D. in Statistics

Academic Editor

PLOS One

Journal Requirements:

If the reviewer comments include a recommendation to cite specific previously published works, please review and evaluate these publications to determine whether they are relevant and should be cited. There is no requirement to cite these works unless the editor has indicated otherwise.

Please review your reference list to ensure that it is complete and correct. If you have cited papers that have been retracted, please include the rationale for doing so in the manuscript text, or remove these references and replace them with relevant current references. Any changes to the reference list should be mentioned in the rebuttal letter that accompanies your revised manuscript. If you need to cite a retracted article, indicate the article’s retracted status in the References list and also include a citation and full reference for the retraction notice.

Additional Editor Comments:

Please review Figure 2 and 3, since some (portions of the) curves fall outside the panel

[Note: HTML markup is below. Please do not edit.]

Reviewers' comments:

Reviewer's Responses to Questions

**Comments to the Author**

1. If the authors have adequately addressed your comments raised in a previous round of review and you feel that this manuscript is now acceptable for publication, you may indicate that here to bypass the “Comments to the Author” section, enter your conflict of interest statement in the “Confidential to Editor” section, and submit your "Accept" recommendation.

Reviewer #4: All comments have been addressed

**********

2. Is the manuscript technically sound, and do the data support the conclusions?

The manuscript must describe a technically sound piece of scientific research with data that supports the conclusions. Experiments must have been conducted rigorously, with appropriate controls, replication, and sample sizes. The conclusions must be drawn appropriately based on the data presented.

Reviewer #4: Yes

**********

3. Has the statistical analysis been performed appropriately and rigorously?

Reviewer #4: Yes

**********

4. Have the authors made all data underlying the findings in their manuscript fully available?

The PLOS Data policy requires authors to make all data underlying the findings described in their manuscript fully available without restriction, with rare exception (please refer to the Data Availability Statement in the manuscript PDF file). The data should be provided as part of the manuscript or its supporting information, or deposited to a public repository. For example, in addition to summary statistics, the data points behind means, medians and variance measures should be available. If there are restrictions on publicly sharing data—e.g. participant privacy or use of data from a third party—those must be specified.

Reviewer #4: Yes

**********

5. Is the manuscript presented in an intelligible fashion and written in standard English?

PLOS ONE does not copyedit accepted manuscripts, so the language in submitted articles must be clear, correct, and unambiguous. Any typographical or grammatical errors should be corrected at revision, so please note any specific errors here.

Reviewer #4: Yes

**********

6. Review Comments to the Author

Please use the space provided to explain your answers to the questions above. You may also include additional comments for the author, including concerns about dual publication, research ethics, or publication ethics. (Please upload your review as an attachment if it exceeds 20,000 characters)

Reviewer #4: The authors have improved the content of the revised manuscript. All my comments/ suggestions have been diligently addressed by the authors and I therefore recommend for the acceptance of the manuscript.

**********

7. PLOS authors have the option to publish the peer review history of their article (what does this mean?). If published, this will include your full peer review and any attached files.

If you choose “no”, your identity will remain anonymous but your review may still be made public.

**Do you want your identity to be public for this peer review?**  For information about this choice, including consent withdrawal, please see our Privacy Policy.

Reviewer #4: No

**********

[NOTE: If reviewer comments were submitted as an attachment file, they will be attached to this email and accessible via the submission site. Please log into your account, locate the manuscript record, and check for the action link "View Attachments". If this link does not appear, there are no attachment files.]

To ensure your figures meet our technical requirements, please review our figure guidelines: https://journals.plos.org/plosone/s/figures

You may also use PLOS’s free figure tool, NAAS, to help you prepare publication quality figures: https://journals.plos.org/plosone/s/figures#loc-tools-for-figure-preparation.

NAAS will assess whether your figures meet our technical requirements by comparing each figure against our figure specifications.

# Author comment: Author response to Decision Letter 3

**DOI:** 10.1371/journal.pone.0331050.r006

11 Jun 2026

Dear Professor Alessandro Barbiero,

Thank you for your valuable comment regarding Figures 2 and 3.

We have carefully revised both figures to ensure that all curves are fully contained within the plotting region. The axis limits have been adjusted appropriately so that no portion of any curve extends outside the frame. The updated figures now clearly display all results without overlap or truncation.

We hope the revised figures now meet the journal’s presentation standards.

Kind regards,

Dr. Bilal Himmat (on behalf of all authors)

Attachment Submitted filename: Response to the editors comments.pdf

# Editor report: Decision Letter 3

**DOI:** 10.1371/journal.pone.0331050.r007

**Authors:** Alessandro Barbiero

16 Jun 2026

A flexible exponential type family for modeling non-monotonic hazard rates with application to mortality analysis : A COVID-19 case study

PONE-D-25-43305R3

Dear Dr. Himmat,

We’re pleased to inform you that your manuscript has been judged scientifically suitable for publication and will be formally accepted for publication once it meets all outstanding technical requirements.

Within one week, you’ll receive an e-mail detailing the required amendments. When these have been addressed, you’ll receive a formal acceptance letter and your manuscript will be scheduled for publication.

An invoice will be generated when your article is formally accepted. Please note, if your institution has a publishing partnership with PLOS and your article meets the relevant criteria, all or part of your publication costs will be covered. Please make sure your user information is up-to-date by logging into Editorial Manager at Editorial Manager® and clicking the ‘Update My Information' link at the top of the page. For questions related to billing, please contact billing support.

If your institution or institutions have a press office, please notify them about your upcoming paper to help maximize its impact. If they’ll be preparing press materials, please inform our press team as soon as possible -- no later than 48 hours after receiving the formal acceptance. Your manuscript will remain under strict press embargo until 2 pm Eastern Time on the date of publication. For more information, please contact onepress@plos.org.

Kind regards,

Alessandro Barbiero, Ph.D. in Statistics

Academic Editor

PLOS One

Additional Editor Comments (optional):

Tha manuscript is now ready for publication.

Reviewers' comments:

# Editor report: Acceptance letter

**DOI:** 10.1371/journal.pone.0331050.r008

**Authors:** Alessandro Barbiero

PONE-D-25-43305R3

PLOS One

Dear Dr. Himmat,

I'm pleased to inform you that your manuscript has been deemed suitable for publication in PLOS One. Congratulations! Your manuscript is now being handed over to our production team.

At this stage, our production department will prepare your paper for publication. This includes ensuring the following:

* All references, tables, and figures are properly cited

* All relevant supporting information is included in the manuscript submission,

* There are no issues that prevent the paper from being properly typeset

You will receive further instructions from the production team, including instructions on how to review your proof when it is ready. Please keep in mind that we are working through a large volume of accepted articles, so please give us a few days to review your paper and let you know the next and final steps.

Lastly, if your institution or institutions have a press office, please let them know about your upcoming paper now to help maximize its impact. If they'll be preparing press materials, please inform our press team within the next 48 hours. Your manuscript will remain under strict press embargo until 2 pm Eastern Time on the date of publication. For more information, please contact onepress@plos.org.

You will receive an invoice from PLOS for your publication fee after your manuscript has reached the completed accept phase. If you receive an email requesting payment before acceptance or for any other service, this may be a phishing scheme. Learn how to identify phishing emails and protect your accounts at https://explore.plos.org/phishing.

If we can help with anything else, please email us at customercare@plos.org.

Thank you for submitting your work to PLOS ONE and supporting open access.

Kind regards,

PLOS ONE Editorial Office Staff

on behalf of

Dr. Alessandro Barbiero

Academic Editor

PLOS One
